# Apaf-1 is an evolutionarily conserved DNA sensor that switches the cell fate between apoptosis and inflammation

**DOI:** 10.1038/s41421-024-00750-4

**Published:** 2025-01-21

**Authors:** Jie Ruan, Xuxia Wei, Suizhi Li, Zijian Ye, Linyi Hu, Ru Zhuang, Yange Cao, Shaozhou Wang, Shengpeng Wu, Dezhi Peng, Shangwu Chen, Shaochun Yuan, Anlong Xu

**Affiliations:** 1 Guangdong Key Laboratory of Pharmaceutical Functional Genes, MOE Key Laboratory of Gene Function and Regulation, MOE Engineering Center of South China Sea Marine Biotechnology, Southern Laboratory of Ocean Science and Engineering (Zhuhai), State Key Laboratory of Biocontrol, School of Life Sciences, Sun Yat-sen University, Guangzhou, Guangdong, China; 2 Laboratory for Marine Biology and Biotechnology, Qingdao National Laboratory for Marine Science and Technology, Qingdao, Shandong China; 3 Sun Yat-sen University Institute of Advanced Studies Hong Kong, Hong Kong, China; 4 School of Life Sciences, Beijing University of Chinese Medicine, Beijing, China

**Keywords:** NOD-like receptors, Stress signalling

## Abstract

Apoptotic protease activating factor 1 (Apaf-1) was traditionally defined as a scaffold protein in mammalian cells for assembling a caspase activation platform known as the ‘apoptosome’ after its binding to cytochrome *c*. Although Apaf-1 structurally resembles animal NOD-like receptor (NLR) and plant resistance (*R*) proteins, whether it is directly involved in innate immunity is still largely unknown. Here, we found that Apaf-1-like molecules from lancelets, fruit flies, mice, and humans have conserved DNA sensing functionality. Mechanistically, mammalian Apaf-1 recruits receptor-interacting protein 2 (RIP2, also known as RIPK2) via its WD40 repeat domain and promotes RIP2 oligomerization to initiate NF-κB-driven inflammation upon cytoplasmic DNA recognition. Furthermore, DNA binding of Apaf-1 determines cell fate by switching the cellular processes between intrinsic stimuli-activated apoptosis and inflammation. These findings suggest that Apaf-1 is an evolutionarily conserved DNA sensor and may serve as a cell fate checkpoint, which determines whether cells initiate inflammation or undergo apoptosis by distinct ligand binding.

## Introduction

The innate immune system has developed several evolutionarily conserved strategies to eliminate infections and to resolve sterile tissue damage that rely on the recognition of molecular patterns present in the invading nonself or abnormal self^1^. For example, Toll-like receptors (TLRs) are known transmembrane pattern recognition receptors (PRRs) that detect a variety of pathogen-associated molecular patterns (PAMPs) to activate the NF-κB signaling pathway from fruit flies to mammals^2^. However, in the field of innate antiviral immunity, diverse antiviral strategies have emerged across species. Plants and invertebrate cells predominantly utilize RNA interference (RNAi) for antiviral defense, while vertebrate cells mainly use cytosolic PRRs, including retinoic acid-inducible gene I (RIG-I)-like receptors (RLRs) and DNA sensors, to recognize foreign or self-nucleic acids, leading to the production of type I interferons (IFNs) and proinflammatory cytokines^3^. In cytoplasmic DNA recognition, several DNA sensors, such as the DNA-dependent activator of interferon regulatory factors (DAI, also known as ZBP1), pyrin and HIN domain (PYHIN) family protein absent-in-melanoma 2 (AIM2), gamma-interferon-inducible protein 16 (IFI16) and cyclic GMP-AMP (cGAMP) synthase (cGAS), have been identified in mammals^4^. Most of these sensors were reported to function via the key adaptor protein stimulator of interferon genes (STING, also called MPYS, MITA, or ERIS) for downstream signaling, but now only the cGAS-STING pathway is thought to be critical for IFN production in response to intracellular DNA and DNA viruses^5^. Evolutionary and structural studies have revealed that both cGAS and STING are very ancient genes, emerging from the sea anemone to the unicellular choanoflagellates *Monosiga brevicollis* and even bacteria^6^. Although bacterial cGAS-like enzymes also synthesize a diverse range of cyclic dinucleotides (CDNs), which further bind to and activate STING homologs after phage infection, bacterial cGAS and the majority of invertebrate cGAS-like receptors (cGLRs) cannot bind DNA^7–11^, suggesting that the DNA-sensing function of cGAS may not be conserved. Indeed, recent studies of *Drosophila melanogaster* cGLR1 and cGLR2 have shown that cGLR1 senses double-stranded RNA (dsRNA) and cGLR2 is activated by an unknown stimulus, but none of them has responsiveness to single-stranded DNA (ssDNA) or dsDNA^12,13^. Moreover, the PYHIN family and their DNA-binding HIN domain exclusively exist in mammals, and AIM2 has even become a pseudogene in several species, such as cow, dog and sheep^14^. Therefore, a critical question is, in a broad evolutionary context, whether the conserved DNA sensors that activate the innate immune responses exist from invertebrates to vertebrates, thereby sensing and clearing unwanted cytoplasmic DNA and maintaining tissue homeostasis physiologically and immunologically.

To answer the above question, we performed comparative immunology studies between vertebrates and lancelets, the most basal living chordates harboring extraordinarily complex innate immunity^15,16^, and surprisingly discovered Apaf-1-like molecules from fruit flies, lancelets, and mammals as novel dsDNA receptors. The Apaf-1 family in metazoans is characterized by a subtype of nucleotide-binding domain (NBD) called nucleotide-binding, Apaf-1, Resistance, and CED4 (NB-ARC) domain^17^. This domain is shared by various plant resistance (*R*) genes that specifically recognize pathogen-derived effectors and form a higher-order complex called the resistosome, triggering a form of programmed cell death at the site of infection known as hypersensitive response (HR)^18^. Other well-characterized NBD-containing proteins are animal NLRs, which directly or indirectly sense diverse PAMPs and host-derived damage-associated molecular patterns (DAMPs) to activate innate immunity. Upon ligand perception, NLRs oligomerize through their NBD and then interact with the adaptor protein apoptosis-associated speck-like protein containing a caspase recruitment domain (ASC) and procaspase-1 to form a large protein complex known as the inflammasome^19^. The recruitment of procaspase-1 to the inflammasome leads to its activation and thereby cleaves its substrates pro-IL-1β and gasdermin D to induce proinflammatory necrotic cell death pyroptosis^20–22^. Although Apaf-1, R proteins, and NLRs have similar domain architectures and are derived from a common ancestor of the ATPase superfamily^23^, until now, the mechanism of mammalian Apaf-1 activation has mainly focused on the surveillance of cytosolic cytochrome *c* (Cyt *c*), which is released from damaged mitochondria and triggers apoptosome assembly and subsequent procaspase-9 activation, resulting in apoptotic cell death^24,25^. Here we discovered Apaf-1-like molecules as evolutionarily conserved dsDNA receptors across species and further showed that Apaf-1 activates NF-κB-dependent expression of proinflammatory cytokines and chemokines by recruiting the adaptor protein RIP2 upon cytoplasmic DNA recognition. Furthermore, a series of cell-free and cell-based assays found that DNA and Cyt *c* compete for Apaf-1 binding to switch the cell fate between apoptosis and inflammation. Thus, this study is the first report highlighting the biological importance of Apaf-1-like molecules in DNA sensing and cell fate determination, which may be a new target for disease therapy, such as viral infection, cancer and autoimmune diseases.

## Results

### Identification of Apaf-1-like molecules as dsDNA receptors across species

**Fig. 1:**
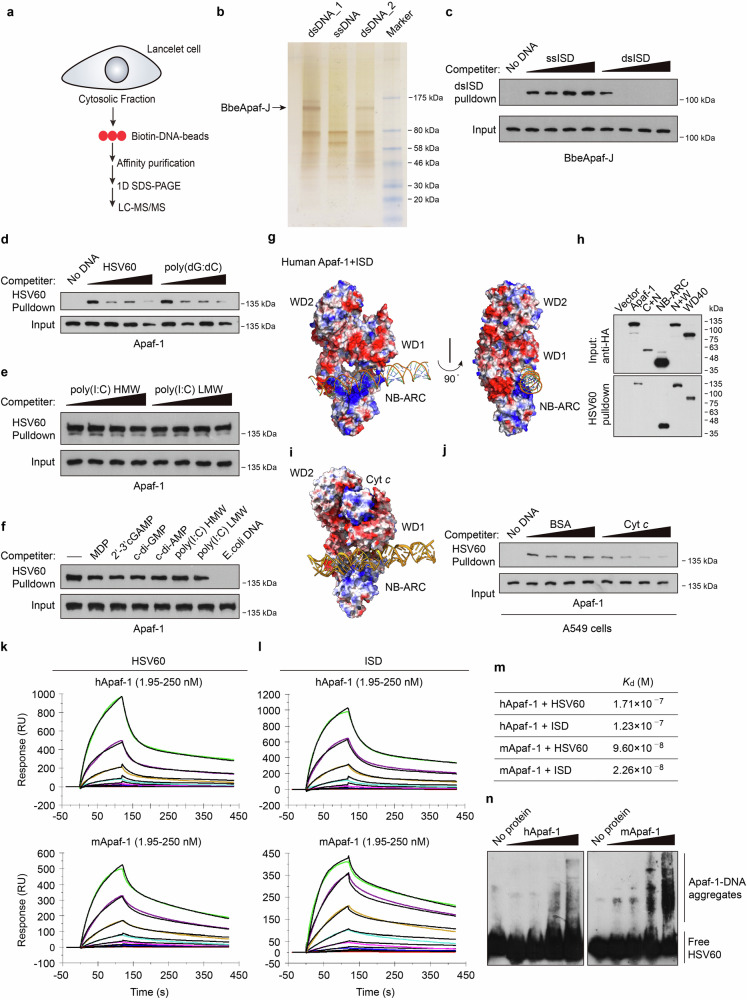
Proteomic screens identify Apaf-1-like molecules as novel DNA receptors. **a** Schematic representation of the mass spectrometry approach to identify DNA binding proteins in lancelet intestinal cells. **b** Silver-stained SDS‒PAGE of precipitated proteins from biotinylated dsISD or ssISD agaroses. The indicated bands were excised and subjected to nano liquid chromatography‒mass spectrometry (nano-LC‒MS). Two dsDNA lines indicate samples from two separate experiments. **c** Competition assays using Flag-BbeApaf-J in transfected HEK293T cell lysates in the presence of increasing amounts (0, 5, 25, 100 μg/mL) of uncoupled ssISD or dsISD and then incubated with biotinylated dsISD agaroses. After incubation, the bound material was analyzed by immunoblotting. **d**–**f** Pull-down competition assays using HA-Apaf-1 in transfected HEK293T cell lysates in the presence of increasing amounts (0, 5, 25, 100 μg/mL) of uncoupled HSV60, poly (dG:dC) (**d**), poly(I:C) HMW or LMW (**e**) and then incubated with biotinylated dsHSV60 agarose. The indicated unlabeled agonists (10 μg/mL) were also used for competition in **f**. **g** Docking structures of the interaction of human Apaf-1 (PDB: 3JBT) with dsISD. Surface electrostatics of the structures was calculated using APBS in PyMol and is color-coded as −59 (red) to +59 (blue) kT/e. Blue and red, positive and negative charge, respectively. **h** Full-length or truncated human Apaf-1 proteins were expressed in HEK293T cells and then incubated with streptavidin beads with biotin-HSV60. Bound proteins were analyzed by immunoblotting with anti-HA antibodies. **i** Superposition of predicted structure of human Apaf-1 bound to dsISD and the Apaf-1: Cyt *c* complex (PDB: 3JBT). **j** Streptavidin pull-down assays of DNA binding to endogenous Apaf-1 with increasing amounts (0, 5, 25, 100 μg/mL) of BSA or purified Cyt *c*. Lysates of A549 cells were incubated with BSA or Cyt *c* for 2 h, and then incubated with biotinylated dsHSV60 agarose. After incubation, the bound material was analyzed by immunoblotting. **k, l** The sensorgrams of chip-immobilized HSV60 and ISD binding to different concentrations of human or mouse Apaf-1 proteins (color lines) are shown, which are expressed in response unit (RU) vs time after subtracting the control signal. Black lines are from model fits. The concentrations of Apaf-1 proteins were 1.95, 3.9, 7.8, 15.6, 31.2, 62.5, 125, 250 nM (from bottom to top). **m** The calculated dissociation constants (*K*_d_) for Apaf-1 proteins. **n** Electrophoretic mobility shift assay (EMSA) of purified human and mouse Apaf-1 proteins at increasing amounts (0, 25, 50, 100, 200 ng) with a high concentration of dsHSV60 (60 fmol). Data are depicted as one representative experiment out of three.

To systematically discover previously unknown DNA sensors in invertebrates, we designed a proteomic screen using DNA affinity purification in lancelet *Branchiostoma belcheri* (Fig. 1a). We first cultured lancelet primary intestine cells, as the digestive system is thought to be the major immune organs of lancelets and contains many immune cells, including lymphocyte-like, monocyte and macrophage-like cells^26^. Previously, we performed a similar screening of poly(I:C)-binding proteins based on lancelet primary cells and identified three evolutionarily conserved dsRNA sensors, DHX9, DHX15 and DDX23^27^. In this study, we coupled biotinylated double-stranded interferon stimulatory DNA (ISD) or its single-stranded counterparts to streptavidin beads to capture DNA receptors from cytosolic extracts of lancelet intestinal cells. The proteins bound to beads were boiled and separated by SDS‒PAGE, followed by silver staining (Fig. 1b). The protein bands were digested by trypsin and analyzed by nano LC‒MS/MS. As expected, we found several well-characterized DNA binding proteins, such as c-Jun and HMGB1, which confirmed the validity and reliability of our screen (Supplementary Table S1).

In addition to HMGB1, we also found a protein that has novel domain architecture, including two N-terminal CARDs fused to a centrally located NB-ARC domain followed by a C-terminal region with no defined domains (Supplementary Fig. S1a, b), and we designated this protein as BbeApaf-J. With the sequencing and annotation of the lancelet *B. belcheri* genome, surprisingly expanded repertoires of Apaf-1 homologs in lancelets have been identified, which not only have one homolog with the classical CARD-NB-ARC-WD40 domain architecture similar to mammalian Apaf-1 but also contain several proteins with novel domain combinations^28^. Alignment analysis of NB-ARC domains revealed that lancelet Apaf-1-like molecules have the consensus nucleotide-binding motifs (Walker A box and Walker B box) similar to other invertebrate and vertebrate homologs of Apaf-1 (Supplementary Fig. S1c). To validate the binding of BbeApaf-J to DNA, we cloned BbeApaf-J from the lancelet *Branchiostoma belcheri* (Bbe) cDNA library. After overexpression of BbeApaf-J in HEK293T cells, whole-cell extracts were prepared and incubated with ssISD or dsISD resin. Evidence for the specific binding of BbeApaf-J to DNA was strengthened by efficient competition with increasing amounts of dsISD (Fig. 1c). The discovery of BbeApaf-J as a novel dsDNA-binding protein inspired our interest in whether this dsDNA-binding capability is evolutionarily conserved.

To test this hypothesis, human Apaf-1 was overexpressed in HEK293T cells, and then the cell lysates were used for a series of pull-down assays. By performing competition experiments, we found that increasing amounts of 60 bp dsDNA from the herpes simplex virus 1 genome (HSV60) or poly(dG:dC) but not the dsRNA analog low molecular weight (LMW) or high molecular weight (HMW) poly(I:C) efficiently blocked the binding of biotin-HSV60 agarose to human Apaf-1 (Fig. 1d, e). To further determine the binding specificity of Apaf-1, several unlabeled agonists, including muramyl dipeptide (MDP), cyclic dinucleotides, poly(I:C) and *E. coli* genomic DNA, were added to HSV60 pull-down assays, but only *E. coli* DNA efficiently blocked the binding of biotin-labeled DNA to Apaf-1 (Fig. 1f).

To explore the conserved DNA-binding mechanism of Apaf-1 homologs from diverse species, we performed protein–DNA docking analyses using published 3D structures of Apaf-1-like molecules (PDB ID: 3JBT for human Apaf-1, PDB ID: 3SFZ for mouse Apaf-1, PDB ID: 5JUL for *Drosophila* Dark) and the BbeApaf-J full-length structure from AlphaFold2. Modeling studies suggested that Apaf-1-like molecules from fruit fly, mouse, and human contain a positively charged surface between their NB-ARC and WD1 domain, which is a portion of WD40 domain (Fig. 1g; Supplementary Fig. S2a, b). Of note, the majority of surface-exposed positively charged amino acids are in the NB-ARC domain, suggesting that the NB-ARC domain may be the primary DNA binding domain of Apaf-1. Interestingly, a similar pocket with positive charge was also found between the NB-ARC and C-terminal (CT) domain of BbeApaf-J (Supplementary Fig. S2c). Consistently, HSV60 pull-down assays using a panel of human Apaf-1 and BbeApaf-J truncated variants found that both the NB-ARC and WD40/CT domains of Apaf-1 or BbeApaf-J bound to DNA (Fig. 1h; Supplementary Fig. S2d). Moreover, the predicted interaction energies of Apaf-1–DNA complex revealed comparable binding affinities from lancelet, fruit fly, mouse, and human (Supplementary Fig. S2e).

In the previously determined atomic structure of the human Apaf-1 apoptosome (PDB ID: 3JBT)^29^, Cyt *c* is sandwiched by the WD1 and WD2 domains of Apaf-1 with perfect shape and charge complementarity, because the charge distribution on the inner face of WD1 and WD2 domains is negatively charged and Cyt *c* is a positively charged proteins (Fig. 1i). We speculate that the conformational changes of Apaf-1 after Cyt *c* binding such as the upward of WD1 in order to fully bind Cyt *c* may enlarge the above-mentioned positively charged pocket and thereby inhibit DNA binding of Apaf-1. Indeed, purified Cyt *c* proteins significantly inhibited the interaction of Apaf-1 with biotinylated DNA (Fig. 1j). Thus, the coordinated DNA binding between NB-ARC and WD40/CT domains in positively charged surface may be the conserved biochemical mechanism of Apaf-1-like molecules for cytosolic DNA sensing.

To further investigate whether Apaf-1-like molecules directly bind to dsDNA and whether such binding is of high affinity, we expressed and purified recombinant human, murine Apaf-1, and *Drosophila* Apaf-1 related killer (Dark) proteins from Sf9 insect cells (Supplementary Fig. S2f, g). Both human and mouse Apaf-1 showed a dose-dependent resonance signal and rapid association with dsDNA on surface plasmon resonance (SPR) (Fig. 1k, l). In contrast, a low resonance signal was detected when incubated with poly(I:C) (Supplementary Fig. S2h). The calculated dissociation constants (*K*_d_) between Apaf-1 and dsDNA were in the low nanomolar range (Fig. 1m), indicating that Apaf-1 has a strong and selective binding affinity to dsDNA. The direct interaction of Apaf-1 with dsDNA was also confirmed by electrophoretic mobility shift assays (EMSA). Interestingly, a smear of high-molecular-weight Apaf-1–DNA aggregates appeared in gel shift assays after incubation with higher concentrations of dsDNA (Fig. 1n), suggesting that the recognition of dsDNA induces Apaf-1 protein oligomerization and the formation of large complexes. Moreover, both human and murine Apaf-1 and Dark formed complexes with dsDNA in a sequence-independent manner (Supplementary Fig. S2i, j). We also performed competition experiments using lysates of baculovirus-infected Sf9 cells and found that increasing amounts of natural genomic DNA, such as calf thymus DNA and herring testis DNA (HT-DNA), robustly inhibited the binding of dsVACV70 to murine Apaf-1 or Dark (Supplementary Fig. S2k, l). Taken together, our results indicate that Apaf-1-like molecules from lancelets, fruit flies, mice, and humans have evolutionarily conserved dsDNA-binding functionality, which may represent a new family of DNA sensors that are termed “Apaf-1-like receptors” (ALRs).

### Apaf-1 is an evolutionarily conserved DNA sensor that activates inflammatory responses

**Fig. 2:**
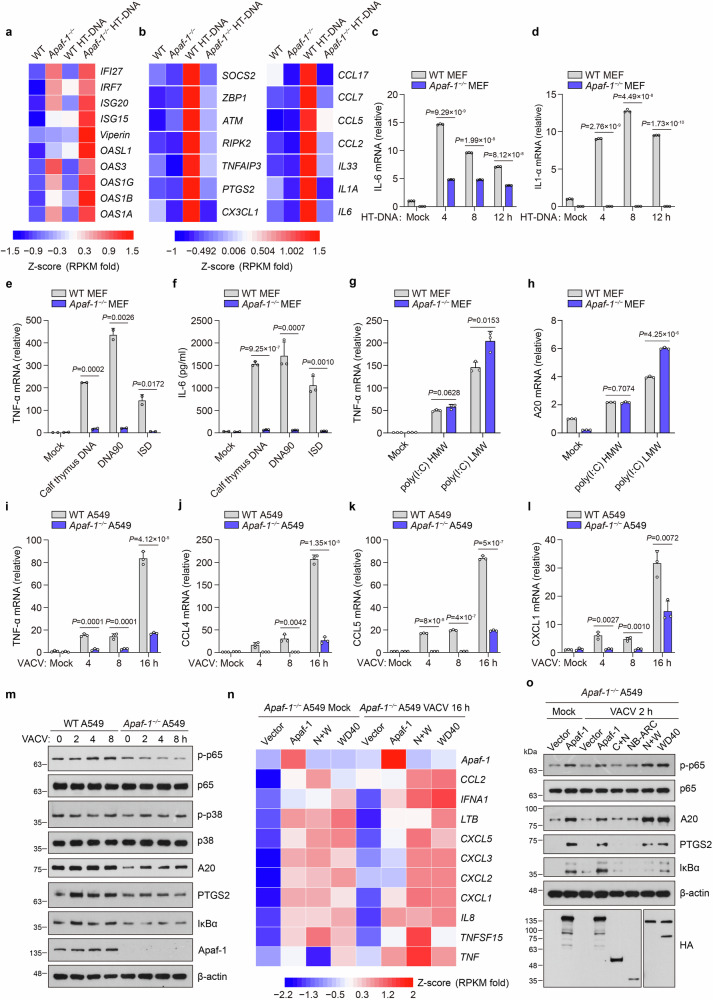
Apaf-1 is an evolutionarily conserved DNA sensor that activates inflammatory responses. **a**, **b** Heatmap of the expression of indicated ISGs (**a**) and inflammation-related genes (**b**) in WT and *Apaf-1*^−/−^ MEFs with or without cytoplasmic HT-DNA transfections (3 μg/mL) for 4 h. **c**, **d** WT and *Apaf-1*^−/−^ MEFs were transfected with HT-DNA (3 μg/mL) at the indicated time points, and the expression of *IL-6* and *IL-1α* was measured by qRT‒PCR. **e**, **f** WT and *Apaf-1*^−/−^ MEFs were transfected with calf thymus DNA, 90-mer dsDNA (DNA90), or ISD (3 μg/mL). The expression of *TNF-α* was measured 12 h later by qRT‒PCR (**e**) while the IL-6 secretion was also quantified by ELISA (**f**). **g**, **h** WT and *Apaf-1*^−/−^ MEFs were transfected with high molecular weight (HMW) poly(I:C) or low molecular weight (LMW) poly(I:C) and the transcription of *TNF-α* and *A20* was measured 12 h later by qRT‒PCR. **i–l** WT and *Apaf-1*^−/−^ A549 cells were infected with VACV (MOI = 3) as indicated and then the *TNF-α, CCL4, CCL5* and *CXCL1* mRNA levels were assayed by qRT‒PCR. **m** Western blotting analysis of the phosphorylation of p65 and p38 and the expression of A20, PTGS2, and IκBα in WT and *Apaf-1*^−/−^ A549 cells infected with VACV for the indicated times. **n** Heatmap of RNA-seq analysis of proinflammatory cytokines and chemokines in *Apaf-1*^−/−^ A549 cells reconstituted with full-length or WD40 repeat domain-containing mutants of Apaf-1 for 24 h and then left untreated (Mock) or infected with VACV for another 16 h. **o** For complementation, HA-tagged full-length or truncated human Apaf-1 proteins were expressed in *Apaf-1*^−/−^ A549 cells for 24 h and then infected with VACV for another 2 h. Western blotting analysis of NF-κB signaling activation is indicated. Data in **m**, **o** are representative of three independent experiments with similar results. Mean ± SEM of triplicates, *P* values were calculated using a two-tailed unpaired Student’s *t-*test.

Since ALRs from fruit flies to humans directly bind to dsDNA, we next explored whether Apaf-1 participates in DNA-triggered innate immune responses in mammals by performing an RNA-seq analysis to compare the transcriptional changes induced by HT-DNA in wild-type (WT) and *Apaf-1*^−/−^ murine embryonic fibroblasts (MEFs). Consistent with previous studies showing that intrinsic apoptosis deficiency induces the IFN response by mitochondrial DNA (mtDNA)-dependent activation of the cGAS-STING pathway in basal or virus infection conditions^30,31^, our results showed the constitutive expression of numerous IFN-stimulated genes (ISGs) in *Apaf-1*^−/−^ MEFs compared to WT MEFs in the resting state. Moreover, the expression levels of those ISGs were highly increased in *Apaf-1*^−/−^ MEFs after HT-DNA stimulation (Fig. 2a). However, we also found another interesting phenomenon that several proinflammatory cytokines, chemokines and inflammation-related genes were markedly reduced in Apaf-1-deficient MEFs after HT-DNA transfection (Fig. 2b). Quantitative RT‒PCR (qRT‒PCR) assays confirmed that transfected HT-DNA activated a potent proinflammatory response and that transcription of *IL-6* and *IL-1α* was reduced when the expression of Apaf-1 was deficient (Fig. 2c, d). Moreover, this Apaf-1-dependent inflammatory response seems not to rely on DNA sequences because the induced transcription of *TNF-α* mRNA and IL-6 protein production by various transfected DNAs derived from different sources, including purified calf thymus DNA, 90-mer dsDNA (DNA90) and ISD, were markedly inhibited in Apaf-1-deficient MEFs (Fig. 2e, f). In contrast, knockout of Apaf-1 in MEFs augmented the expression of *TNF-α* and the primary NF-κB response gene *A20* (also known as *tumor necrosis factor α-induced protein 3, TNFAIP3*) induced by LMW or HMW poly (I:C) transfections (Fig. 2g, h), which is consistent with a previous study showing that the intrinsic apoptotic pathway negatively regulated dsRNA and RNA virus-triggered immune responses^32^. To further validate the role of Apaf-1, *Apaf-1*^−/−^ A549 cells were generated by CRISPR-Cas9-mediated targeting. Consistently, the absence of Apaf-1 in A549 cells significantly blocked the DNA virus vaccinia virus (VACV)-triggered expression of NF-κB-controlled cytokines and chemokines, including *TNF-α*, *CCL4 (*also known as *macrophage inflammatory protein 1-beta, MIP-1β)*, *CCL5 (RANTES)* and *CXCL1 (KC)* (Fig. 2i–l). Moreover, both the phosphorylation of p65 and the expression of NF-κB target genes such as A20, PTGS2/Cox2 and IκBα were also compromised in *Apaf-1*^−/−^ A549 cells compared with WT cells after VACV infection (Fig. 2m), suggesting that Apaf-1 has an unexpected role in cytosolic DNA or DNA virus-induced inflammatory responses in both mouse and human cells.

To comprehensively understand the pro-inflammatory function of Apaf-1, we conducted RNA-seq assays and compared the gene expression of *Apaf-1*^−/−^ A549 cells reconstituted with human Apaf-1 and control cells. The re-expression of Apaf-1 in *Apaf-1*^−/−^ A549 cells activated the expression of several pro-inflammatory cytokines, chemokines and NF-κB target genes in resting states and after VACV infection (Fig. 2n, o). Interestingly, WD40 repeat domain-containing Apaf-1 mutants robustly induced the VACV-induced phosphorylation of p65 and expression of A20, PTGS2, and IκBα, whereas mutants expressing CARD + NB-ARC or the NB-ARC domain alone could not (Fig. 2n, o). Gene ontology (GO) and Kyoto Encyclopedia of Genes and Genomes (KEGG) pathway enrichment demonstrated that the differentially expressed genes were significantly enriched in immune responses against viruses (Supplementary Fig. S3a–f), indicating that the WD40 repeat domain of Apaf-1 has an essential role in initiating NF-κB-driven inflammation upon DNA virus infection. Taken together, our results reveal that Apaf-1 is an evolutionarily conserved DNA sensor to activate the proinflammatory NF-κB pathway.

### Apaf-1 mediates the activation of NF-κB induced by DNA virus infection in a cGAS- and STING-independent manner

To provide the direct evidence that Apaf-1 binds viral DNA upon DNA virus infection, we first overexpressed Apaf-1 into HEK293T cells and then performed immunoprecipitation and qRT–PCR assays. Both Apaf-1 and cGAS showed high affinity for the herpes simplex virus 1 (HSV-1) DNA whereas the well-known dsRNA sensor RIG-I did not (Supplementary Fig. S4a–d). A similar assay also detected the binding ability of endogenous Apaf-1 to HSV-1 DNA in A549 cells (Supplementary Fig. S4e).

**Fig. 3:**
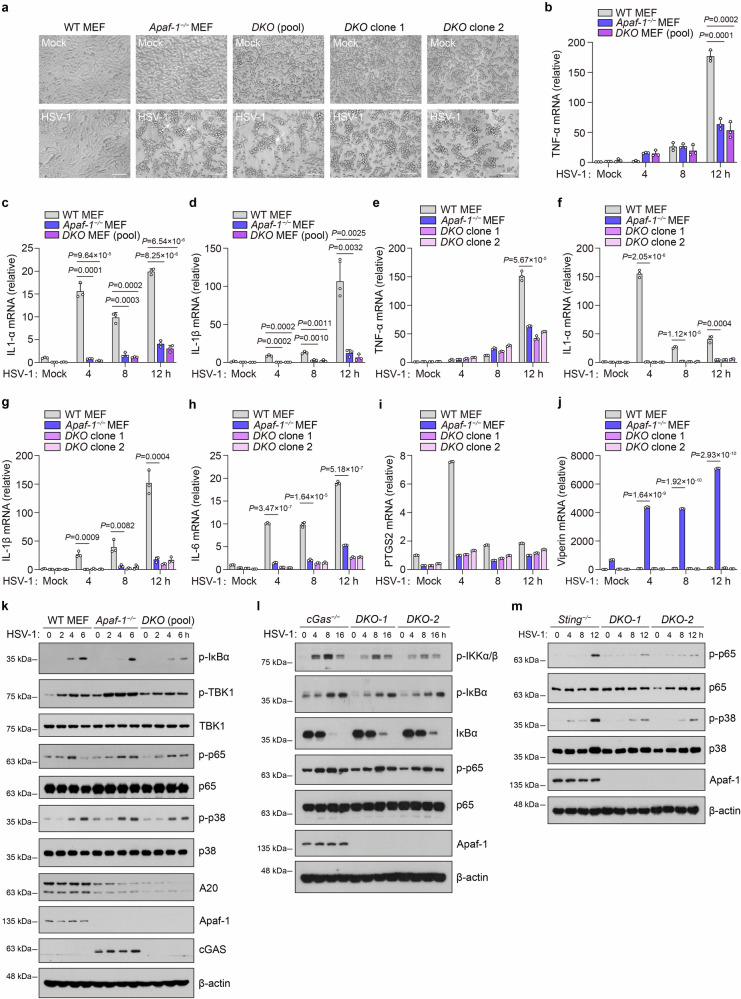
Apaf-1 mediates DNA virus infection-induced inflammatory responses independently of cGAS and STING. **a** WT, *Apaf-1*^−/−^ MEFs, pooled *Apaf-1*^−/−^*cGas*^−/−^ (*DKO*) MEF cells, and two *DKO* clones were mock-infected or infected with HSV-1 (MOI = 1.5) and analyzed 12 h later. Their morphology was observed by phase-contrast microscopy. Scale bars, 100 μm. **b**–**d** WT, *Apaf-1*^−/−^ MEFs, and pooled *Apaf-1*^−/−^*cGas*^−/−^ MEF cells were infected with HSV-1 (MOI = 1.5) at the indicated time points, and the expression of *TNF-α*, *IL-1α*, and *IL-1β* was measured by qRT–PCR. **e**–**j** WT, *Apaf-1*^−/−^ MEFs, and two DKO clones were infected with HSV-1 (MOI = 1.5) at the indicated time points, and the expression of *TNF-α*, *IL-1α*, *IL-1β*, *IL-6*, *PTGS2*, and *Viperin* was measured by qRT–PCR. **k** Western blot analysis of the phosphorylation of IκBα, p65 and TBK1 and the expression of the NF-κB target gene A20 in WT, *Apaf-1*^−/−^ and pooled *Apaf-1*^−/−^*cGas*^−/−^ MEFs infected with HSV-1 (MOI = 3) for the indicated time points. **l** Western blotting analysis of the phosphorylation of IKKα/β, IκBα, p65, and IκBα degradation in *cGas*^−/−^ and *cGas*^−/−^*Apaf-1*^−/−^ (*DKO*) HeLa clone 1 and clone 2 infected with HSV-1 (MOI = 3) at the indicated time points. **m** Western blotting analysis of the phosphorylation of p65 and p38 in *Sting*^−/−^ and *Sting*^−/−^*Apaf-1*^−/−^ (*DKO*) HeLa clone 1 and clone 2 infected with HSV-1 (MOI = 3) at the indicated time points.

Given that the cGAS-STING axis is considered the major sensing pathway for cytosolic DNA, we next investigated whether Apaf-1-mediated NF-κB signaling is dependent on cGAS and STING. We generated *Apaf-1*^−/−^*cGas*^−/−^ MEF cells through CRISPR–Cas9-mediated targeting and infected WT, *Apaf-1*^−/−^ and *Apaf-1*^−/−^*cGas*^−/−^ MEF cells with HSV-1. Consistent with the phenomenon occurred in *Apaf-1*^−/−^ MEF cells, both pooled *Apaf-1*^−/−^*cGas*^−/−^ MEF cells and two *DKO* clones displayed profound morphological changes such as cell rounding, clumping and detachment after HSV-1 infection (Fig. 3a), suggesting that they are more sensitive to HSV-1 infection compared with their WT counterparts. More importantly, we found dramatic decreases in HSV-1-induced expression of pro-inflammatory cytokine in both pooled *Apaf-1*^−/−^ *cGas*^−/−^ MEF cells and two *DKO* clones to a similar degree as previously shown in *Apaf-1*^−/−^ MEF cells (Fig. 3b–i). In contrast, the constitutive and HSV-1-induced expression of *Viperin* (a well-known ISG) in *Apaf-1*^−/−^ MEF cells was completely dependent on cGAS (Fig. 3j). These data clearly indicate that the essential role of Apaf-1 in DNA virus-induced inflammatory responses is independent of cGAS.

It has been demonstrated that cGAS utilizes interferon regulatory factor 3 (IRF3) and NF-κB pathways to exert its effects^33^. We investigated whether the downstream signaling of Apaf-1 induced by HSV-1 infection involves the activation of these transcription factors. WT, *Apaf-1*^−/−^, and pooled *Apaf-1*^−/−^*cGas*^−/−^ MEF cells were infected with HSV-1 as indicated, and total cell extracts were prepared and analyzed by SDS‒PAGE followed by western blotting assay. We observed that either HSV-1 or RNA virus vesicular stomatitis virus (VSV) infection dramatically increased the phosphorylation of TBK1 and p38 in *Apaf-1*^−/−^ MEFs, and their levels were decreased in *Apaf-1*^−/−^*cGas*^−/−^cells (Fig. 3k; Supplementary Fig. S4f). This phenomenon is consistent with previous studies showing that the intrinsic apoptosis deficiency caused by knocking out Apaf-1 augments the IFN response in basal or viral infection conditions via the mtDNA-dependent cGAS-STING activation^30,31^. However, the phosphorylation of IκBα and p65 after HSV-1 infection, which is the hallmark of its activation, was reduced in *Apaf-1*^−/−^ MEFs at early time points (2 h and 4 h) post-infection compared with WT cells. Moreover, induction of the NF-κB target gene A20 was diminished in both *Apaf-1*^−/−^ and *Apaf-1*^−/−^*cGas*^−/−^ MEF cells (Fig. 3k). Interestingly, we also found that the levels of p-IκBα and p-p65 still increased at late time points (6 h) in both *Apaf-1*^−/−^ and *Apaf-1*^−/−^*cGas*^−/−^ MEF cells, suggesting that other DNA sensors may be activated by HSV-1 infection in the absence of Apaf-1 and cGAS (Fig. 3k).

To further confirm the above results, we generated *cGas*^−/−^*Apaf-1*^−/−^ and *Sting*^−/−^*Apaf-1*^−/−^ HeLa cells through the CRISPR–Cas9-mediated targeting and found that HSV-1-induced NF-κB activation including the phosphorylation of IKKα/β, IκBα, p65 and IκBα degradation was significantly inhibited in *cGas*^−/−^*Apaf-1*^−/−^ (*DKO*) HeLa clones (Fig. 3l). In addition, we also observed that the HSV-1 infection-triggered phosphorylation of p65 and p38 was greatly reduced in *Sting*^−/−^ cells at early time points (4 h and 8 h), which confirm the essential role of STING in innate immune responses against DNA virus infection^34,35^. However, p65 and p38 phosphorylation still increased at 12 h post-infection in *Sting*^−/−^ cells and the knockout of Apaf-1 inhibited HSV-1-induced p-p65 and p-p38 (Fig. 3m).

Besides, we further explored whether Apaf-1 activates NF-κB signaling independently of cGAS and STING through the gain-of-function approach. We transfected plasmids encoding HA-tagged Apaf-1 into HEK293T cells, which do not express cGAS, STING, and PYHIN proteins. The overexpression of Apaf-1 in HEK293T cells significantly increased HSV-1 but not the RNA virus VSV-induced p65 phosphorylation (Supplementary Fig. S4g, h). Moreover, the accumulation of the HSV-1 protein infected cell protein 27 (ICP27) was decreased in Apaf-1-overexpressing cells compared to HEK293T cells transfected with control vectors at the early time point (4 h) after HSV-1 infection (Supplementary Fig. S4g), indicating that the activation of NF-κB by Apaf-1 induces host defense against DNA viruses in the absence of cGAS and STING. Consistently, we also found that the presence of phosphorylated p65 in the nuclear fraction of HEK293T cells after HSV-1 infection was substantially elevated in Apaf-1-overexpressing cells by subcellular fractionation analysis (Supplementary Fig. S4i). In addition, the overexpression of Apaf-1 also resulted in enhanced p65 phosphorylation in response to dsHSV60 (Supplementary Fig. S4j), suggesting that Apaf-1 activates NF-κB responses independently of cGAS and STING upon DNA virus or cytosolic DNA stimulation.

In addition, we transfected control, cGAS, or Apaf-1 expression vectors into *cGas*^−/−^ HeLa cells and then tested HSV-1-induced signaling profiles. We observed that HSV-1-induced phosphorylation of TBK1 was strictly dependent on cGAS, as ectopic expression of cGAS restored TBK1 activation and *Viperin* expression. However, the phosphorylation of p65 and p38 in response to HSV-1 infection did not rely on cGAS but on Apaf-1, which augmented HSV-1-mediated NF-κB activation and the induction of proinflammatory cytokines such as *TNF-α* in cGAS-deficient cells (Supplementary Fig. S4k–m). Moreover, the overexpression of Apaf-1 in *cGas*^−/−^ HeLa cells increased the activation of NF-κB-driven luciferase induced by HSV-1 infection in a dose-dependent manner (Supplementary Fig. S4n). Taken together, our results show that Apaf-1 is independent of cGAS and STING in triggering NF-κB activation in response to cytoplasmic DNA or DNA virus infection.

### Caspase-9 is dispensable for DNA virus infection-induced inflammatory responses

Given that the well-described function of Apaf-1 is to induce apoptosis by recruiting and activating caspase-9 after its binding to Cyt *c*, we generated *Casp9*^−/−^ MEF cells using CRISPR‒Cas9-mediated targeting to investigate whether caspase-9 contributes to DNA virus-induced inflammatory responses. Both WT and *Casp9*^−/−^ MEF cells displayed similar morphological changes, such as cell rounding and most of them still adhered to culture surfaces after HSV-1 infection, which is distinct from the phenotype observed in *Apaf-1*^−/−^ MEFs (Fig. 3a; Supplementary Fig. S5a). Consistently, caspase-9 deficient cells displayed equal or greater capacity to induce inflammatory responses compared with WT cells after HSV-1 infection (Supplementary Fig. S5b–f). We also found the increased expression of *Viperin* in *Casp9*^−/−^ MEF cells compared to WT cells after HSV-1 infection (Supplementary Fig. S5g), which is consistent with previous studies showing that the apoptotic caspase deficiency augments the IFN response in basal or virus infection conditions^30,31^. Consistent with the expression of proinflammatory cytokines and chemokines, the phosphorylation of p65 and the expression of the NF-κB target gene A20 following HSV-1 infection were comparable between WT and caspase-9-deficient cells (Supplementary Fig. S5h). These data collectively suggest that caspase-9, another key component of the apoptosome, is dispensable for DNA virus infection-induced inflammatory responses. Thus, Apaf-1-mediated inflammatory signaling is independent of the intrinsic apoptotic pathway.

### Apaf-1 is required for DNA virus- or DNA-induced inflammation in mice

**Fig. 4:**
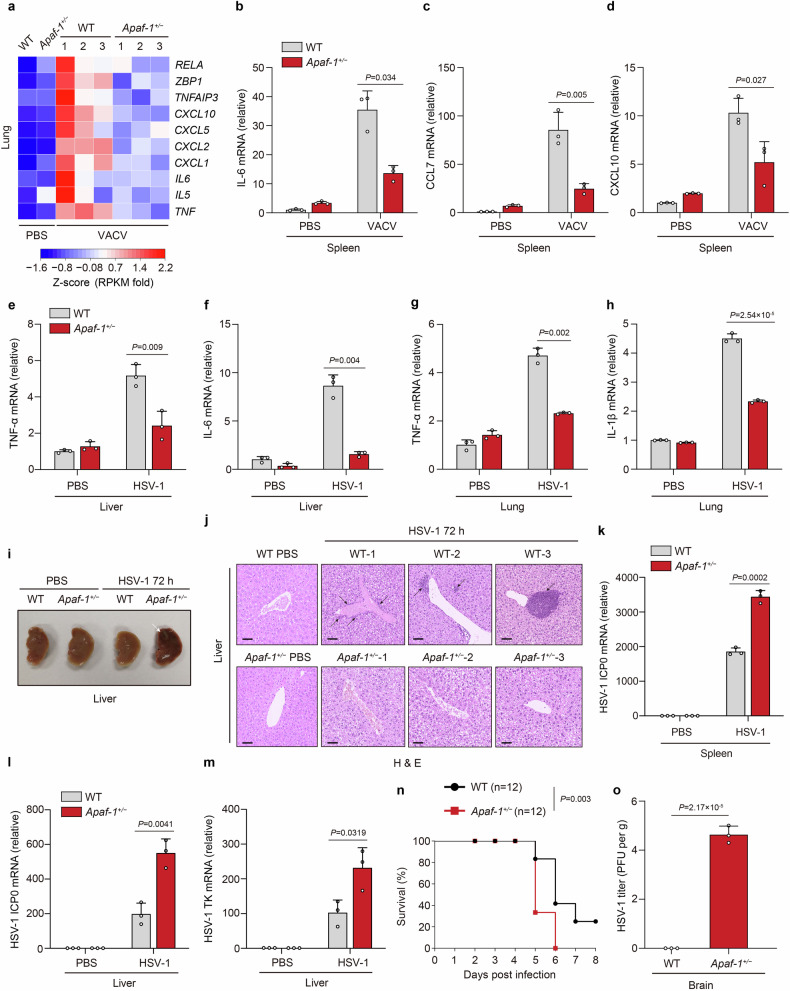
Apaf-1 is required for inflammation against DNA virus infection in mice. **a** Heatmap of proinflammatory cytokines, chemokines, and NF-κB target genes in the lungs of WT and *Apaf-1*^+/−^ mice infected with VACV (2 × 10^7^ PFU per mouse) for 16 h (*n* = 3 mice per group). **b**–**d** WT mice and their *Apaf-1*^+/−^ littermates (*n* = 3 mice per group) were injected with VACV intraperitoneally (2 × 10^7^ PFU per mouse) for 16 h, followed by measurement of *IL-6*, *CCL7*, and *CXCL10* mRNAs in livers and lungs. **e**–**h** *Apaf-1*^+/−^ mice and their WT littermates (*n* = 3 mice per group) were injected intravenously with HSV-1 (1 × 10^7^ PFU per mouse) for 24 h, followed by measurement of *TNF-α*, *IL-6*, or *IL-1β* mRNA levels in livers and lungs. **i** Representative images of livers from WT and *Apaf-1*^+/−^ mice uninfected and infected with HSV-1 (1 × 10^7^ PFU per mouse) for 72 h. The white arrow indicates HSV-1-induced liver lesions or viral hepatitis. **j** Hematoxylin & eosin staining of liver tissues from three different WT and *Apaf-1*^+/−^ mice infected with HSV-1 (1 × 10^7^ PFU per mouse) for 72 h. Scale bars, 100 μm. Black arrows indicate inflammation marked by leukocyte infiltration. **k**–**m** The qRT‒PCR analysis of HSV-1 *ICP0* or *TK* mRNAs in spleens and livers from WT and *Apaf-1*^+/−^ (*n* = 3 per group) 8-week-old mice infected with HSV-1 (1 × 10^7^ PFU per mouse) for 72 h. **n** Survival of WT and *Apaf-1*^+/−^ mice (*n* = 12 per group) after intranasal infection with HSV-1 (5 × 10^7^ PFU per mouse). **o** HSV-1 titers in the brains of WT and *Apaf-1*^+/−^ mice (*n* = 3 per group) infected with HSV-1 (2 × 10^7^ PFU per mouse) for 72 h. Viral titers were determined by the plaque assay. Mean ± SEM of triplicates, *P* values were calculated using two-tailed unpaired Student’s *t-*test in **a**–**h** and **k**–**m** and **o**. The log-rank (Mantel‒Cox) test was used in **n**.

As the absence of functional intrinsic apoptosis causes potent antiviral responses even at the basal state, which can complicate the phenotype, we employed an Apaf-1 heterozygous knockout (*Apaf-1*^+/−^) mouse model to further explore the physiological role of Apaf-1 in vivo (Supplementary Fig. S6a–c). Heterozygous *Apaf-1*^+/−^ mice were viable and without obvious defects, as previously reported^36,37^. Eight-week-old WT and *Apaf-1*^+/−^ mice were infected with VACV via the intraperitoneal route. Substantial reductions in proinflammatory cytokines and chemokines were observed in Apaf-1 heterozygous knockout lungs through RNA sequencing (RNA-seq) analysis (Fig. 4a). Moreover, differentially expressed genes of WT vs *Apaf-1*^+/−^ lungs were significantly enriched in innate immunity, such as Herpes simplex virus 1 infection, NOD-like receptor signaling pathway and TNF signaling pathway (Supplementary Fig. S6d). This phenotype was further verified by qRT‒PCR analysis, which detected the decreased expression of *IL-6*, *CCL7* (also known as *monocyte chemotactic protein 3, MCP-3*), *CXCL10* (also known as *interferon (IFN)-γ-induced protein 10, IP-10*) and *CXCL1* in the spleens or lungs of *Apaf-1*^+/−^ mice after VACV infection (Fig. 4b–d; Supplementary Fig. S6e–g). To confirm this result and exclude viral type specificity, we intravenously infected WT and *Apaf-1*^+/−^ mice with HSV-1. The reduced expression of *TNF-α*, *IL-6*, *IL-1β* and *CXCL1* was also observed in the livers and lungs of *Apaf-1*^+/−^ mice after HSV-1 infection compared with that of WT mice (Fig. 4e–h; Supplementary Fig. S6h). In contrast, the HSV-1-induced transcription of *Viperin* in the lungs was comparable between WT and *Apaf-1*^+/−^ mice (Supplementary Fig. S6i). Moreover, we found profound pathological alterations in the livers of *Apaf-1*^+/−^ mice at 72 h post-infection, including dark color and significant virus-induced liver lesions or viral hepatitis (Fig. 4i). Histological analyses from three different WT and *Apaf-1*^+/−^ mice displayed HSV-1 infection-induced inflammation marked by leukocyte infiltration in the liver, which was conspicuously absent in *Apaf-1*^+/−^ mice (Fig. 4j), suggesting that Apaf-1-mediated inflammatory responses are essential for efficient protection against HSV-1 infection. Indeed, enhanced HSV-1 replication in the spleens and livers of *Apaf-1*^+/−^ mice was determined by measuring the expression of two key HSV-1-encoded genes, *ICP0* and *TK* (Fig. 4k–m). Furthermore, WT and *Apaf-1*^+/−^ mice were infected intranasally with HSV-1, and survival was monitored. Most of the *Apaf-1*^+/−^ mice died on Day 5 after infection, while 80% of WT mice survived (Fig. 4n). Significant amounts of HSV-1 were detected in the brains of *Apaf-1*^+/−^ mice but not in WT mice 3 days after infection (Fig. 4o). To determine the role of Apaf-1 in immune defense against RNA virus infection in vivo, we infected WT and *Apaf-1*^+/−^ mice with VSV via the intravenous route. ELISA analyses showed that the sera of WT and *Apaf-1*^+/−^ mice contained comparable levels of TNF-α and IL-6 after VSV infection (Supplementary Fig. S6j, k). In addition, we investigated whether Apaf-1 is required for cytosolic DNA-triggered signaling in primary immune cells. Bone marrow-derived macrophages (BMDMs) from WT and *Apaf-1*^+/−^ mice were isolated and transfected with HT-DNA. Compared to their WT counterparts, *Apaf-1*^+/−^ BMDMs exhibited reduced expression of *IL-6* at the early time point (4 h) after HT-DNA transfections but had similar increases in *IFN-β* and *Viperin* transcription (Supplementary Fig. S6l–n). Collectively, these data indicate that Apaf-1 has an important role in host defense against DNA virus infection in vivo, and this physiological relevance is independent of the intrinsic apoptosis deficiency.

### Apaf-1 recruits RIP2 to mediate inflammatory responses upon cytoplasmic DNA sensing

**Fig. 5:**
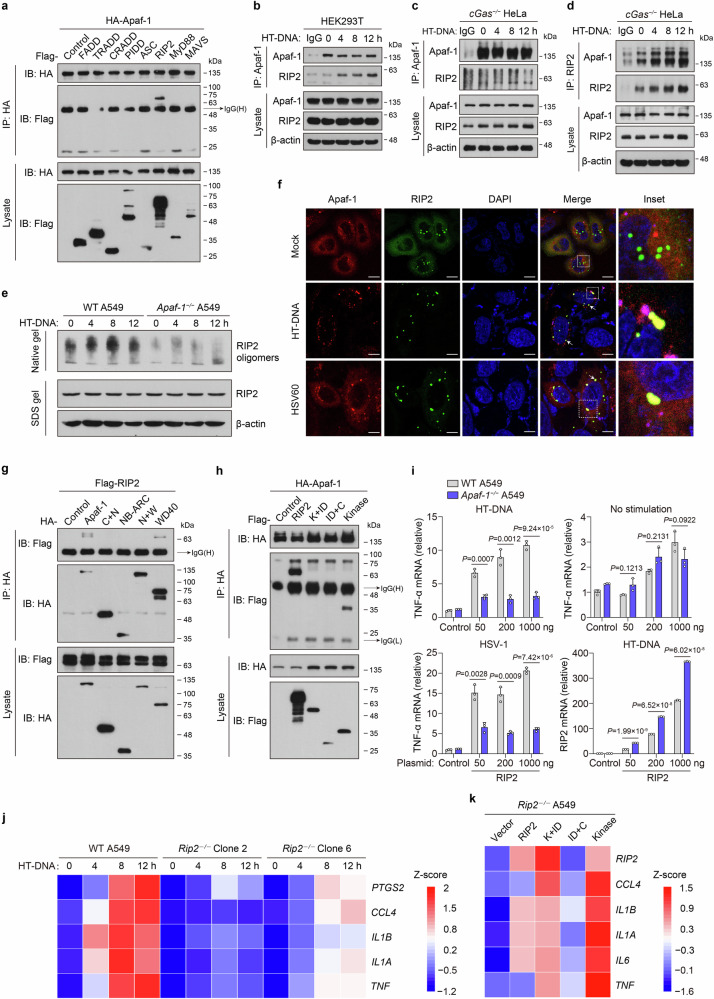
Apaf-1 recruits RIP2 to mediate inflammatory responses upon cytoplasmic DNA sensing. **a** HEK293T cells were co-transfected with HA-tagged Apaf-1 and plasmids expressing Flag-tagged proteins containing death domains (DD). Proteins were immunoprecipitated with anti-HA antibody, and bound proteins were detected with anti-Flag or anti-HA antibody. **b**–**d** Protein complex of Apaf-1 and RIP2 in HEK293T and *cGas*^−/−^ HeLa cells, which were transfected with HT-DNA (3 μg/mL) for the indicated times. Immunoblot analysis of endogenous Apaf-1 and RIP2 immunoprecipitated with antibodies against Apaf-1 or RIP2. **e** WT and *Apaf-1*^−/−^ A549 cells were transfected with HT-DNA as indicated. Cell lysates were separated by native PAGE or SDS‒PAGE and analyzed by immunoblotting with the indicated antibodies. **f** The *cGas*^−/−^ HeLa cells expressing both mCherry-Apaf-1 and EGFP-RIP2 were transfected with HT-DNA or HSV60. At 4 h post-transfection, cells were fixed, stained with DAPI, and imaged by confocal microscopy. Scale bars, 10 μm. **g** HA-tagged full-length or truncated Apaf-1 expression plasmids were coexpressed with Flag-tagged RIP2 in HEK293T cells for 24 h and then infected with HSV-1 for another 4 h. After incubation with anti-HA beads, the bound proteins were analyzed by immunoblotting with anti-Flag or anti-HA antibodies. **h** Flag-tagged RIP2 or truncated expression plasmids were coexpressed with HA-tagged Apaf-1 in HEK293T cells for 24 h and then transfected with HT-DNA for another 4 h. After incubation with anti-HA beads, the bound proteins were analyzed by immunoblotting with anti-HA or anti-Flag antibodies. **i** Expression plasmids (50, 200, and 1000 ng) of Flag-tagged human RIP2 were transfected into WT or *Apaf-1*^−/−^ A549 cells for 24 h and then treated with HT-DNA (3 μg/mL) or HSV-1 (MOI = 3) for 12 h. The expression of *TNF-α* or *RIP2* was measured by qRT‒PCR. **j** Heatmap of the induction of *TNF-α*, *IL-1α*, *IL-1β*, *CCL4*, and *PTGS2* mRNA in WT and two different *Rip2*^−/−^ A549 cells transfected with HT-DNA (3 μg/mL) for the indicated times. **k** Heatmap of indicated pro-inflammatory cytokine and chemokine expression profiles in full-length or truncated RIP2 reconstitution in *Rip2*^−/−^ A549 cells with HT-DNA stimulation for 12 h. Mean ± SEM of triplicates, *P* values were calculated using a two-tailed unpaired Student’s *t-*test.

We next investigated the mechanisms by which Apaf-1 mediates DNA virus- or cytoplasmic DNA-triggered immune responses. Apaf-1 contains an amino (N)-terminal caspase-activation and recruitment domain (CARD) and is supposed to be activated by a CARD-containing protein. We first examined eight mammalian death domain proteins, which all have crucial roles in cell death and inflammation, to identify possible Apaf-1-interacting proteins. Surprisingly, only receptor-interacting protein 2 (RIP2, also known as RICK, CARDIAK, and RIPK2)^38–40^ could interact with Apaf-1 upon HSV-1 infection (Fig. 5a). Consistently, the expression level of *RIP2/RIPK2* was also significantly increased in WT MEFs upon HT-DNA stimulation, and Apaf-1 deficiency nearly abolished this upregulation (Fig. 2b). These observations suggest that RIP2 may be the downstream target of Apaf-1, which mediates cytosolic DNA or DNA virus-induced inflammation. Our previous results have found that the WD40 domain of Apaf-1 mediated proinflammatory NF-κB activation upon DNA virus infection (Fig. 2n, o). We therefore performed an immunoprecipitation–mass spectrometry (IP-MS) experiment in *Apaf-1*^−/−^ A549 cells reconstituted with HA-tagged NB-ARC + WD40 or HA-tagged WD40 domain to further confirm above co-IP screen results. Although high baseline ISG expression levels of *Apaf-1*^−/−^ A549 cells may contribute to several unspecific interactions in the top hits, these experiments not only successfully identified Cyt *c* (CYCS) as WD40 domain-binding proteins, but also revealed that RIP2 potentially interacted with the WD40 domain of Apaf-1 after VACV infections. Interestingly, we also found NF-κB1 and NF-κB2, two key NF-κB family members in the results, confirming the involvement of WD40 repeat domain in DNA virus-induced NF-κB signaling (Supplementary Fig. S7a). Moreover, the endogenous Apaf-1/RIP2 complex was detected in HEK293T cells and *cGas*^−/−^ HeLa cells, and their association was augmented in response to HT-DNA transfection (Fig. 5b–d).

RIP2 is the well-known downstream adaptor of the bacterial peptidoglycan sensors NOD1 and NOD2. It was reported that ligand-activated NOD1/NOD2 oligomerize and associate with RIP2, triggering proinflammatory signaling pathways and leading to the activation of NF-κB and mitogen-activated protein kinases (MAPKs)^41^. To investigate the mechanism underlying how Apaf-1 interacts and induces RIP2 activation in response to cytoplasmic DNA, we first utilized chemical cross-linkers to stabilize protein complexes in lysates from primary mouse ear fibroblasts transfected with HT-DNA or ISD. Interestingly, cytoplasmic DNA treatment increased the endogenous oligomeric forms of RIP2 ( > 180 kDa) in a time-dependent manner (Supplementary Fig. S7b). Moreover, HT-DNA-induced RIP2 oligomerization was decreased in *Apaf-1*^−/−^ A549 cells under both native and cross-linking conditions (Fig. 5e; Supplementary Fig. S7c), indicating that Apaf-1 is required for DNA-stimulated oligomerization of RIP2. We further examined the cellular state of Apaf-1 and RIP2 proteins by confocal microscopy using HeLa cells expressing mCherry-Apaf-1 and EGFP-RIP2. In the absence of a stimulus, the majority of Apaf-1 and RIP2 proteins were distributed diffusely throughout the cytoplasm, with a few bright RIP2 specks that could be observed. Intriguingly, after the cells were transfected with HT-DNA or dsHSV60 for 4 h, the distribution of Apaf-1 and RIP2 became more aggregated, and they colocalized within large puncta of more intense staining (Fig. 5f).

To further study which domain of Apaf-1 was required for this interaction, we cotransfected a series of truncated mutants of Apaf-1 and RIP2 into HEK293T cells. Domain mapping experiments indicated that either full-length or WD40 domain interacted with RIP2 after HSV-1 infection (Fig. 5g), which was consistent with the abovementioned functional analysis (Fig. 2n, o) and indicated that the WD40 repeat domain of Apaf-1 mediates proinflammatory NF-κB activation by interacting with the adaptor protein RIP2. On the other hand, a series of RIP2 truncated mutants were co-expressed with full-length Apaf-1 in HEK293T cells (Supplementary Fig. S7d). Interestingly, co-immunoprecipitation assays showed that full-length RIP2 and the kinase domain of RIP2 significantly interacted with Apaf-1 (Fig. 5h). These data indicate that Apaf-1 recruits RIP2 through the interaction of WD40-kinase domains upon cytoplasmic DNA recognition, which is distinct from the canonical CARD–CARD interaction between Apaf-1 and caspase-9 during the intrinsic apoptotic cell death.

To explore the functional relationship between Apaf-1 and RIP2, different amounts of RIP2 expression plasmid were transfected into WT or *Apaf-1*^−/−^ A549 cells with HT-DNA stimulation or HSV-1 infection. The results showed that HT-DNA or HSV-1 significantly enhanced *TNF-α* induction by RIP2 plasmids in a dose-dependent manner in WT cells (Fig. 5i). In contrast, the overexpression of RIP2 failed to induce *TNF-α* expression in Apaf-1-deficient cells in response to HT-DNA or HSV-1 infection. qRT‒PCR analyses confirmed the robust upregulation of RIP2 in transfected WT and *Apaf-1*^−/−^ A549 cells (Fig. 5i).

To validate the role of RIP2, we generated *Rip2*^−/−^ A549 cells using CRISPR‒Cas9 technology and transfected these cells with HT-DNA (Supplementary Fig. S7e). Intriguingly, the induction of the proinflammatory mediators *TNF-α, IL-1α, IL-1β, CCL4* and *PTGS2* was severely reduced in *Rip2*-deficient cells in a time-dependent manner (Fig. 5j). Moreover, we reintroduced full-length and truncated forms of RIP2 into *Rip2*^−/−^ A549 cells and found that only full-length and kinase domain-containing RIP2 mutants could restore HT-DNA-induced expression of proinflammatory cytokines and chemokines (Fig. 5k), confirming that RIP2 plays a critical role in cytosolic DNA-induced inflammatory responses and highlighting the functional importance of its kinase domain in mediating NF-κB activation.

Given that RIP2 was originally classified as a serine-threonine kinase based on homology scans performed in the 1990s and autophosphorylation is the major mechanism of RIP2 activation in the NOD1/2 signaling pathway^42,43^, we next used either kinase dead or catalytically inactive mutant of RIP2 (K47A and D146N) to test whether RIP2 kinase activity is required for Apaf-1 signaling. RIP2-deficient cells reconstituted with the RIP2 K47A or D146N mutant displayed equal or greater capacity to induce inflammatory responses compared with cells expressing WT RIP2 after HT-DNA transfections (Supplementary Fig. S7f–i). In addition, RIP2 kinase dead mutation did not prevent the Apaf-1–RIP2 interaction after HT-DNA transfections (Supplementary Fig. S7j, k). Thus, these data suggest that the kinase activity of RIP2 is not required for Apaf-1-RIP2 signaling and we hypothesized that the kinase domain of RIP2 may use distinct mechanisms to mediate the Apaf-1-induced signaling. To verify this hypothesis, we constructed *Rip2*^−/−^ A549 cells stably expressing EGFP-tagged full-length or truncated mutants of RIP2 to perform confocal microscopy experiments. Under mock-transfected conditions, the majority of full-length and the kinase domain-containing RIP2 mutants (K + ID and Kinase) were distributed diffusely throughout the cytoplasm, with a few bright aggregates of the CARD domain-containing mutants (ID + C). This phenomenon is consistent with previous studies showing that RIP2CARD spontaneously forms protein filaments in vitro and in cells^44,45^. Interestingly, after HT-DNA transfections for 12 h, the distribution of full-length RIP2, RIP2 K + ID and kinase mutants became more aggregated, and they colocalized with Apaf-1 in several large speck-like structures. In contrast, a majority of RIP2 CARD still remain distributed diffusely in response to HT-DNA (Supplementary Fig. S7l–o). Collectively, these results are consistent with our previous data performed in *cGas*^−/−^ HeLa and HEK293T cells (Fig. 5f, h) and suggest that upon cytoplasmic DNA sensing, Apaf-1 recruits RIP2 via the RIP2 kinase domain, which may further facilitate the aggregation of RIP2 protein and formation of Apaf-1/RIP2 complexes.

### DNA or DNA virus infection switches Cyt *c*-mediated apoptotic cell death to inflammation by competing with Apaf-1 binding

**Fig. 6:**
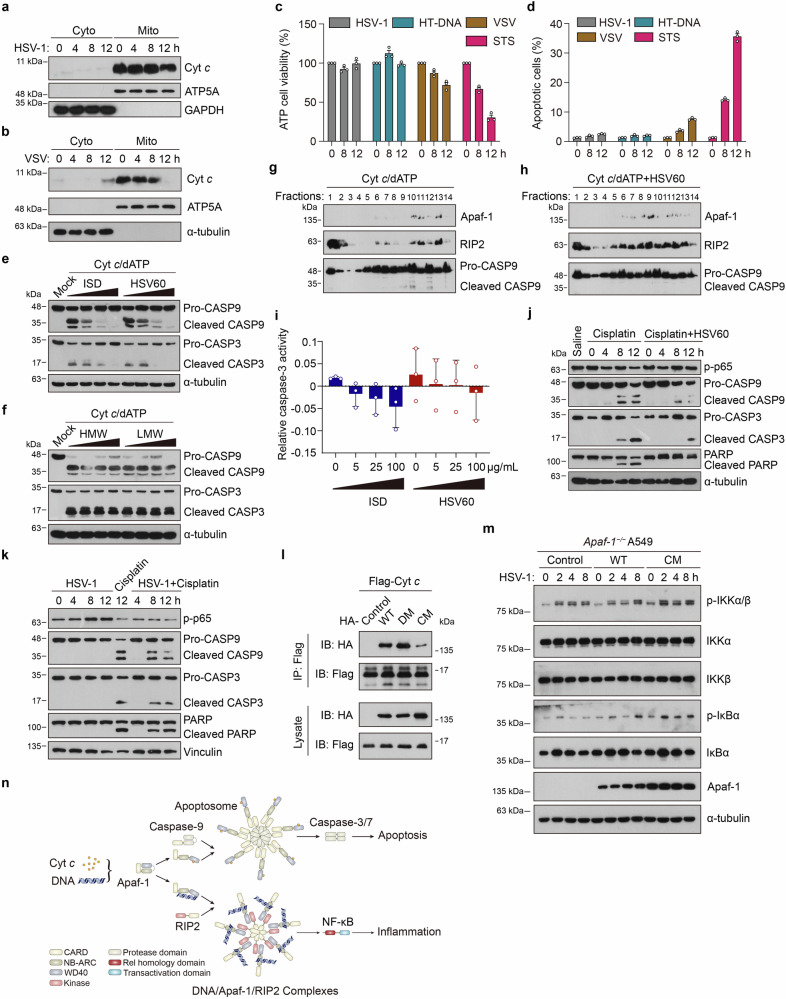
Cytosolic DNA or DNA virus infection switches Cyt *c* and chemotherapy drug-induced apoptosis to inflammation by competing with Apaf-1 binding. **a** WT MEFs were infected with HSV-1 (MOI = 3) for the indicated hours and then subjected to digitonin fractionation followed by immunoblot analysis. GAPDH and ATP5A serve as cytosolic and mitochondrial markers, respectively. **b** WT MEFs were infected with VSV (MOI = 3) for the indicated hours and then subjected to digitonin fractionation followed by immunoblot analysis. α-tubulin and ATP5A serve as cytosolic and mitochondrial markers, respectively. **c** MEFs were treated with HSV-1 (MOI = 3), HT-DNA (3 μg/mL), VSV (MOI = 3), or staurosporine (STS) as indicated, and then the cell survival rates were determined by measuring ATP levels. **d** MEF cells were treated with HSV-1 (MOI = 3), HT-DNA (3 μg/mL), VSV (MOI = 3) or staurosporine (STS) as indicated and then stained with propidium iodide and annexin V-FITC for flow cytometry. The proportions of apoptotic cells are summarized. **e** A549 cell-free extracts were treated with increasing amounts (0, 25, 50, 100 μg/mL) of ISD or HSV60 for 2 h and then incubated with 50 μg/mL Cyt *c* and 1 mM dATP as indicated. After incubation, the reaction mixture was analyzed by immunoblotting. **f** A549 cell-free extract was also treated with increasing amounts (0, 25, 50, 100 μg/mL) of poly (I:C) HMW or LMW for 2 h and then incubated with 50 μg/mL Cyt *c* and 1 mM dATP as indicated. After incubation, the reaction mixture was analyzed by immunoblotting. **g**, **h** A549 cell-free extracts were incubated with 50 μg/mL Cyt *c* and 1 mM dATP in the absence (**g**) or presence (**h**) of 100 μg/mL of dsHSV60 at 30 °C for 2 h. After incubation, the reaction mixture was subjected to glycerol gradient ultracentrifugation (10% –45%). Fractions were collected and immunoblotted with an Apaf-1, RIP2, or caspase-9 antibody as indicated. **i** A549 cell-free extracts were treated with increasing amounts (0, 25, 50, 100 μg/mL) of ISD or HSV60 for 2 h and then incubated with 50 μg/mL Cyt *c*, 1 mM dATP and substrates of caspase-3 Ac-DEVD-pNA. After incubation, the caspase-3 activity in the reaction mixture was measured. **j** The *cGas*^−/−^ HeLa cells were treated with vehicle (saline), cisplatin, or cisplatin + HSV60 for the indicated times, and the phosphorylation of p65 and the processing of caspase-9, caspase-3, and PARP were analyzed by immunoblotting. **k** The *cGas*^−/−^ HeLa cells were infected with HSV-1 (MOI = 3) for the indicated hours (4, 8, 12 h) with or without cisplatin. Cell lysates were analyzed by immunoblotting to assess the phosphorylation of p65 and the processing of caspase-9, caspase-3, and PARP. **l** Full-length HA-tagged wild-type (WT), DNA-binding mutant (DM), or Cyt *c*-binding mutant (CM) of human Apaf-1 proteins were co-expressed with Flag-tagged Cyt *c* in HEK293T cells for 24 h. After incubation with anti-Flag beads, the bound proteins were analyzed by immunoblotting with anti-Flag or anti-HA antibodies. **m** *Apaf-1*^−/−^ A549 cells were reconstituted with WT or Cyt *c*-binding mutant (CM) of Flag-tagged human Apaf-1 and infected with HSV-1 (MOI = 3) for the indicated times. Western blot analysis of the phosphorylation of IKKα/β and IκBα were shown. **n** Proposed working model of the cell fate decision between apoptosis and inflammation by distinct ligand binding of Apaf-1. Upon DNA sensing, Apaf-1 induces NF-κB-driven inflammatory responses by recruiting the adaptor protein RIP2 through the WD40-kinase interaction, which in turn oligomerizes and forms the DNA/Apaf-1/RIP2 complex, thus switching Cyt *c*-activated apoptosis to inflammation. Data are representative of three independent experiments with similar results.

Mammalian Apaf-1 is well known for its binding to Cyt *c* in apoptotic cell death. To further elucidate the relationship between Apaf-1-mediated intrinsic apoptosis and its proinflammatory functions, we investigated whether HSV-1 infection induces Cyt *c* release using biochemical analysis. Up to 12 h after efficient HSV-1 infection in WT MEFs, there was no release or depletion of Cyt *c* from the mitochondria. In contrast to DNA virus HSV-1, VSV dramatically triggered Cyt *c* release from mitochondria to cytosol at 12 h post-infection (Fig. 6a, b; Supplementary Fig. S8a). Consistently, the viability of MEF cells was similar after HT-DNA transfection and HSV-1 infection but dropped significantly following VSV infection and staurosporine (STS) treatment (Fig. 6c). Moreover, cleavage of caspase-3 and its substrate PARP was not detected in either WT or *Apaf-1*^−/−^ MEFs after HSV-1 infection (Supplementary Fig. S8b). We further analyzed MEF cell profiles through fluorescence-activated cell sorting (FACS) and found that most cells were double-negative for annexin V and propidium iodide upon HT-DNA transfection or HSV-1 infection, which proceeded directly to the annexin V-positive and propidium iodide-negative stage after STS treatment (Fig. 6d; Supplementary Fig. S8c). To exclude cell type specificity, we also tried to use A549 cells to confirm this phenomenon. FACS analyses correlated with MEF data and indicated that HSV-1 infection and cytoplasmic DNA did not trigger apoptotic cell death in either MEFs or A549 cells (Supplementary Fig. S8d–f).

To provide direct evidence that Apaf-1 sits at the crossroads between DNA- or DNA virus-induced apoptosis and inflammation, we modified a cell-free system^46^ to study whether exogenous DNA influences Cyt *c-* and dATP-triggered caspase activation. The results showed that the addition of dsISD or dsHSV60 to A549 cell-free extracts significantly inhibited Cyt *c* and dATP-activated caspase-3 and caspase-9 cleavage in a dose-dependent manner regardless of their sequences while adding the synthetic dsRNA analog poly(I:C) did not have this effect (Fig. 6e, f). Moreover, we used glycerol gradient ultracentrifugation (10%–45%) to monitor the interaction between Apaf-1 and RIP2 in the cell-free system. In the presence of Cyt *c* and dATP, the protein peaks of Apaf-1 and cleaved caspase-9 were found at high-molecular-weight fractions 10 and 12, indicating that they formed the apoptosome. In contrast, the majority of RIP2 is present at the top of the gradient, with a small portion of RIP2 co-migrated with the oligomerized Apaf-1 in fractions 11 and 13 but not with cleaved caspase-9 (Fig. 6g). Interestingly, we found that the addition of dsHSV60 to this assay significantly triggered the migration of RIP2 to high-molecular-weight fractions and the protein peaks of Apaf-1–RIP2 interaction were observed at fraction 9, suggesting that they form another large complex after DNA binding. Consistently, dsHSV60 addition dramatically inhibited Cyt *c*/dATP-activated caspase-9 cleavage (Fig. 6h). Furthermore, we also measured caspase-3 activity in the extracts and found that it was decreased with the addition of dsISD or dsHSV60 in a similar dose-dependent manner (Fig. 6i). These results were consistent with our DNA pull-down competition assays (Fig. 1j) and strongly suggested that upon different ligand binding (DNA or Cyt *c*), Apaf-1 switches the cellular fate between inflammation and apoptosis by forming distinct complexes (the Apaf-1/RIP2 complex or apoptosome).

Chemotherapy drugs such as cisplatin are known to kill cells by activating Apaf-1-mediated intrinsic apoptosis. We tested whether HSV-1 or dsHSV60 treatment switched cisplatin-induced apoptosis to inflammation and vice versa in cell-based assays. The dsHSV60 stimulation substantially decreased the cleavage of caspase-9, caspase-3 and PARP upon the treatment with cisplatin (Fig. 6j). Conversely, the cisplatin treatment augmented caspase-9, caspase-3 and PARP cleavage while inhibiting the phosphorylation of p65 in HSV-1-infected HeLa cells (Fig. 6k). Moreover, recent studies have shown that small-scale mitochondrial outer membrane permeabilization (termed minority MOMPs, miMOMPs) can generate low levels of Cyt *c* release, caspase activation and chronic inflammation without culminating in cell death^47,48^. To further confirm the above cell-fate decision is indeed mediated via Apaf-1, we have designed a triple mutation in the previously determined interface between Cyt *c* and WD2 domain of Apaf-1 (W884A/D902A/T1087A)^29^ to test whether loss of Cyt *c* binding increases Apaf-1-mediated inflammatory responses upon DNA virus infections. As expected, co-immunoprecipitation assays showed that the Cyt *c*-binding mutant (CM) of Apaf-1 greatly reduced the ability to interact with Cyt *c* compared with WT and the DNA-binding mutant (DM) (Fig. 6l). Interestingly, we found that Apaf-1-deficient cells reconstituted with the Cyt *c*-binding mutant have greater capacity to trigger NF-κB activation compared with cells expressing WT Apaf-1 after HSV-1 infections by measuring the phosphorylation of IKKα/β and IκBα (Fig. 6m). This phenomenon is consistent with our previous data and further suggests that Apaf-1 has its own gatekeeper of activation: low levels of Cyt *c* in the cytosol caused by miMOMPs may compete with dsDNA for Apaf-1 binding, thus suppressing the pro-inflammatory activity of Apaf-1. In contrast, Apaf-1 mutants lacking the key Cyt *c*-binding residues that are not restricted at this level may have stronger pro-inflammatory functions. Taken together, our analyses suggest a mechanism for regulating cell fate decisions in response to cytoplasmic DNA or DNA virus infection that depends on the ligand specificity of Apaf-1 (Fig. 6n).

## Discussion

It was known for many years that DNA could stimulate innate immune responses before it was known as the carrier of genetic materials in living organisms^49^. However, among eukaryotes, the mechanism of cytoplasmic DNA sensing has thus far only been studied in mammals. In this comparative immunology study, we found that ALRs from lancelets, fruit flies, mice, and humans directly bind to dsDNA. The mechanism of DNA sensing by ALRs provides a new paradigm of Apaf-1 activation. Previous studies have shown that the CED-4/Apaf-1 family is a highly conserved component of intrinsic (or mitochondrial) apoptosis, which is characterized by MOMP and the activation of caspases by Cyt *c*. However, whether Cyt *c* binding is the prerequisite for Apaf-1 activation remains controversial^50^. It was shown that the nematode *C. elegans* Apaf-1 homolog CED-4 can directly activate caspase CED-3 and initiate intrinsic apoptotic cell death without the involvement of MOMP and Cyt *c*^51^. Moreover, the *Drosophila* Apaf-1 homolog Dark forms an apoptosome in the absence of Cyt *c*, which does not bind to the Dark protein in vitro^52^. Thus, our findings suggest that recognition of cytoplasmic DNA is the primordial and conserved function of Apaf-1 from fruit flies to humans, which may predate the emergence of Cyt *c*-mediated intrinsic apoptosis in deuterostomes. The physiological roles of ALR-mediated cytoplasmic DNA sensing beyond pathogen resistance are also intriguing. It was previously shown that the knockout of Apaf-1 or Dark preferentially causes severe developmental defects in the central nervous system (CNS), such as brain overgrowth in mice and hyperplasia of the brain lobes in *Drosophila*^36,37,53^. These predominant phenotypes were commonly assumed only as the result of apoptotic cell death deficiency but cannot properly explain why Apaf-1 knockout has little impact on the embryonic development of other organs and why *Apaf-1*^−/−^ mice have more severe brain abnormalities than *Casp9*^−/−^ or *Casp3*^−/−^ mice^36^. Given that neurodevelopment is characterized by rapid neural cell proliferation resulting in high levels of DNA damage, sensing and cleaning damaged DNA by ALRs may contribute to normal neurodevelopment in both invertebrates and vertebrates. Further investigation of the functional consequence of DNA damage sensing by ALRs will provide new strategies for the treatment of various neurological diseases.

Our structural modeling and biochemical analyses showed that coordinated DNA binding between NB-ARC and WD40/CT domains in positively charged surfaces may be the conserved mechanism of ALRs for cytosolic DNA sensing. Moreover, our complementation studies demonstrated that the expression of the WD40 repeat domain alone is sufficient to activate the inflammatory response after DNA virus infection. The WD40 repeat domain is among the top ten most abundant domains in the eukaryotic genome and is involved in diverse biological processes, including growth, cell cycle control, and transcriptional regulation^54^. To date, no WD40 repeat domain has been identified with innate immune sensing activity. Our data here may provide an interesting initial observation for investigating the role of other WD40 repeat-containing proteins in immune responses and also suggest the conserved role of the C-terminal regions of ALRs in cytoplasmic DNA sensing and signaling. To test this hypothesis, we conducted RNA-seq assays and compared the gene expression of *Apaf-1*^−/−^ A549 cells reconstituted with either full-length or truncated mutants of BbeApaf-J with control cells in response to VACV infection. As shown in our results, elevated type I and type III IFNs, TNFα and other proinflammatory cytokines as well as chemokines were observed in Apaf-1-deficient cells reconstituted with the full-length, 2CARDs + NB-ARC (2C + N) or C-terminal (CT) domain of BbeApaf-J (Supplementary Fig. S9a). Consistent with the above RNA-seq results, the reconstitution of BbeApaf-J-2C + N or CT domain was sufficient to increase the phosphorylation of p65 and expression of NF-κB target genes (Supplementary Fig. S9b). Moreover, KEGG pathway enrichment demonstrated that the differentially expressed genes were significantly enriched in innate immunity (Supplementary Fig. S9c–e). Thus, our results reveal that ALRs in lancelets and humans are evolutionarily conserved DNA sensors that activate the proinflammatory NF-κB pathway.

Although the DNA sensing function of ALRs is highly conserved, some differences in signal transduction may exist between species. We found that BbeApaf-J has an additional CARD domain that is also involved in inflammation-inducing function. Thus, it is likely that the CARD domain of ALRs has evolutionary adaptations from dual roles of proinflammatory/proapoptotic signaling in invertebrates to be specifically involved in the activation of intrinsic apoptosis in vertebrates, reflecting the tuning of inflammation to prevent self-damage and autoimmunity during evolution. Additional studies in other invertebrate animal models such as *Drosophila* may provide a more detailed mechanistic understanding of this process. Moreover, our IP-MS and endogenous IP data indicate that human Apaf-1 recruits the adaptor protein RIP2 after DNA sensing, which belongs to the well-conserved receptor-interacting protein (RIP) family and has the strongest similarity to *Drosophila* Imd (immune deficiency)^55^. In the *Drosophila* innate immunity, Toll and Imd pathways are essential for host defense against fungal and bacterial infections, which share similar functions with the mammalian TLRs and TNF signaling pathways^56^. Given our biochemical experiments in which Dark proteins directly bind to dsDNA in vitro, it will be interesting to explore whether Imd is the downstream partner of Dark, whether and how the Dark-Imd axis activates inflammatory responses in response to cytosolic DNA or DNA virus infection in *Drosophila*. Furthermore, we have performed evolutionary analyses of RIPK family across species during our studies. The phylogenetic trees show that RIP6 and RIP7, also called leucine-rich repeat (LRR) kinase LRRK1 and LRRK2, are the most ancient members and exist in *Drosophila*, Hydra, lancelet and mammals. Of note, RIPK2 homologs in lancelets are expanded and contain proteins with novel domain combinations including LRR-TIR-Kinase, LRR-LRR-Kinase, and LRR-Death-Kinase. This phenomenon is shared by lancelet Apaf-1 homologs and the expansion implied the roles of RIPK2-like molecules in immune responses. Interestingly, we also found two lancelet RIPK2 homologs, solely having a kinase domain (XP_019628684.1 and XP_019626679.1) or one with the Kinase-Death domain composition similar to that of human RIPK2 (XP_019629560.1) (Supplementary Fig. S10a, b). Given that human Apaf-1 interacts with RIPK2 kinase domain to initiate NF-κB-driven inflammation upon cytoplasmic DNA sensing, it is conceivable that BbeApaf-J may recruit these RIP2-like molecules in lancelets to activate NF-κB in response to DNA virus infections, which requires further analysis.

The discovery of Apaf-1 as an evolutionarily conserved DNA sensor significantly raises questions about the role of this molecule in cell fate decisions. Deregulation of intrinsic apoptosis is associated with various pathologic conditions. Excessive apoptosis occurs in strokes and ischemia-reperfusion injuries, and conversely, deficient or inadequate apoptosis is linked to cancer and autoimmunity^57^. Although great efforts have been made, the clinical development of small-molecule inhibitors targeting intrinsic apoptosis is still limited, and inhibiting caspases has largely failed in clinical trials because of severe side effects and unsatisfactory efficacy^58^. This may be due to the nonapoptotic roles of some caspases and targeting caspases that act at the late stage in apoptotic cell death, since this is unlikely to be protective when cells are already destined to die in response to severe damage. Given the centrality of Apaf-1 in the initiation of intrinsic apoptosis, our results propose a new mechanism by which cell fate decisions during stress or infection can be made at the upstream innate sensing stage. In this new paradigm, the ligand specificity of Apaf-1 determines whether cells initiate inflammation or undergo apoptosis (that is, DNA binding of Apaf-1 switches intrinsic stimuli-activated apoptosis to NF-κB-driven inflammation), which may provide another interesting target for developing new therapeutic strategies for intrinsic apoptosis and inflammation-mediated diseases. We believe that it will be promising to deliver dsDNA to the site of ischemic organ injuries such as the heart and brain, to inhibit the Cyt *c* binding of Apaf-1 and lethal apoptotic loss of cardiomyocytes and promote the survival of neurons. Moreover, in the context of neoplasia, tumor cells have evolved various strategies to evade intrinsic apoptosis, such as increasing the expression of antiapoptotic BCL-2 family proteins (BCL-2, BCL-X_L_, and MCL-1) and harboring extensive genome instability and DNA damage during cell division, leading to the accumulation of self-DNA structures, including micronuclei and chromatin fragments in the cytosol^59,60^. Thus, we speculate that binding of this endogenous DNA by Apaf-1, accompanied by impaired Cyt *c* release in tumor cells, may be an intrinsic driving force for tumorigenesis by triggering NF-κB-driven chronic inflammation. Small molecule inhibitors targeting the DNA-binding interface of Apaf-1 may be used in new cancer therapy by combining them with other drugs, such as BH3-mimetics, to switch tumor cell fates from persistent inflammation to apoptotic cell death.

## Materials and Methods

### Mice

*Apaf-1*^+/−^ mice were purchased from GemParmatech and generated by comicroinjection of in vitro translated Cas9 mRNA and gRNAs into C57BL/6J zygotes. All mice were bred and maintained in a specific pathogen-free facility. All animal experiments were conducted in compliance with guidelines established by the Sun Yat-sen University Institutional Animal Care and Use Committee.

### Cell culture and transfection

A549 and HEK293T cells were obtained from ATCC. C57BL/6 J mouse-derived WT and *Apaf-1*^−/−^ MEFs were kindly provided by Dr. Xiaodong Wang (National Institute of Biological Sciences, Beijing). *cGas*^−/−^ and *Sting*^−/−^ HeLa cells were gifted by Dr. Zhengfan Jiang (Peking University). These cells were cultured in Dulbecco’s modified Eagle’s medium (DMEM, Thermo Fisher Scientific) supplemented with 10% FBS (Gibco), 10 mM HEPES and 2 mM L-glutamine. All cells were grown at 37 °C in a 5% CO_2_ incubator and tested to be mycoplasma-negative by PCR. JetPRIME was used to transfect plasmid DNA into HEK293T or HeLa cells by following the manufacturer’s instructions. Bone marrow cells from WT and *Apaf-1*^+/−^ mice were differentiated into macrophages in DMEM (Thermo Fisher Scientific) supplemented with 10% FBS (Gibco) and 20% (v/v) L929-conditioned medium at 37 °C in a 5% CO2 incubator and were collected on Day 7 for experiments.

### Culture of primary lancelet intestinal cells

Adult Chinese lancelet *Branchiostoma belcheri* were collected from Zhanjiang, China, and reared in aerated sea water with algae. Three days before dissection, the lancelets were transferred to seawater without algae to clear their intestine. On the day prior to dissection, seawater supplemented with penicillin (10 mg/mL) was filtered with a 0.22 μm strainer for intestine sterilization.

After the lancelets were anesthetized, the intestines were extracted, dissected into pieces, and digested for 2 h at 23 °C with 1% collagenase. The cells were suspended in culture medium (DMEM high glucose, F12, and Leiboviz’s L15 at a ratio of 1:1:2) containing 10% FBS and penicillin-streptomycin. The cell suspension was cultured in a 35 mm tissue culture dish at a density of five intestines per dish at 23 °C.

### Plasmids and reagents

Complementary DNA (cDNA) for lancelet *BbeApaf-J* was amplified by RT‒PCR amplification and 5’RACE using mRNA from lancelet intestinal tissue as a template. The BbeApaf-J cDNA was then cloned into pcDNA3-Flag. cDNAs for *Apaf-1* and eight death domain-containing genes were obtained from GeneCopoeia and inserted into a modified pReceiver-3× Flag or 3× HA vector for transient expression in HEK293T and HeLa cells. Truncation mutants of Apaf-1 were constructed by the standard PCR cloning strategy and inserted into the pcDNA3 vector with the indicated tags. cGAMP, c-di-GMP, c-di-AMP, low molecular weight (LMW) or high molecular weight (HMW) poly(I:C), poly(dA:dT), and poly(dG:dC) were purchased from InvivoGen. Calf thymus DNA and herring testis DNA (HT-DNA) were purchased from Sigma. ISD or HSV60 were prepared from equimolar amounts of sense and antisense DNA oligonucleotides, which were synthesized at Invitrogen, heated at 95 °C for 5 min, and cooled to room temperature.

The following antibodies were used for immunoprecipitation and/or immunoblotting: anti-Apaf-1 (#ab234436) and anti-cytochrome *c* (#ab110325) were obtained from Abcam. The p-TBK1 (#5483), p-p38 (#4511), p-p65 (#3033), p-IKKα/β (#2078), p-IκBα (#9246), IκBα (#4814), A20 (#5630), PTGS2 (#12282), PARP (#9532), caspase-3 (#9662) and caspase-9 (#9508) antibodies were from Cell Signaling Technology (CST). Anti-HA (#H6533) and anti-Flag (#F1804) beads were purchased from Sigma. Antibodies for GAPDH (#60004-1-Ig), β-actin (#60008-1-Ig), α-tubulin (#66031-1-Ig), ATP5A (#66037-1-Ig), Vinculin (#66305-1-Ig) and Histone H3 (#17168-1-AP) were obtained from Proteintech.

### Isolation of DNA binding proteins from lancelet intestine cells

For the generation of ISD affinity resins, high-capacity streptavidin agarose (Pierce) was washed three times with PBS. Then, biotinylated-ssISD or dsISD was added to washed resins, followed by incubation for 2 h at 4 °C on a rotary wheel to allow binding of biotinylated DNA. Next, ISD resins were washed twice with TAP lysis buffer (20 mM HEPES pH 7.5, 10 mM KCl, 5 mM MgCl_2_, 1 mM EDTA, 1 mM EGTA, 5% (v/v) glycerol, 0.2% NP-40, and protease inhibitors) for the removal of free ISD.

Lancelet primary cells were lysed in TAP lysis buffer, and protein concentrations were measured with a Bradford assay. Cell extracts were added to the ssISD or dsISD resin followed by 12 h at 4 °C. After incubation, resins were washed extensively with lysis buffer, resuspended in SDS sample buffer, separated by SDS‒PAGE, and stained with silver.

### DNA pull-down assay

To test DNA binding to Apaf-1 in cell lysates, HEK293T cells were transfected with the indicated HA-tagged Apaf-1 expression plasmids. Cells were collected and lysed in TAP lysis buffer for 1 h at 4 °C. A total of 10 μg of biotin-labeled HSV60 was immobilized onto 20 μL of streptavidin agarose. Unconjugated DNA was removed by extensively washing with lysis buffer. Cleared lysates were incubated with these agaroses for 1 h at 4 °C. For the competition assay, increasing amounts (0, 5, 25, 100 μg/mL) of free HSV60, poly(dG:dC), poly(I:C) HMW, LMW or 10 μg of unlabeled cGAMP, c-di-GMP, poly(I:C), and *E. coli* DNA were first incubated with cell lysates for 1 h at 4 °C before adding biotin-conjugated HSV60 resins. The resins were washed extensively with lysis buffer and resuspended in SDS‒PAGE sample buffer, followed by western blotting assay. To examine whether Cyt *c* influences the DNA binding of Apaf-1, increasing amounts (0, 5, 25, 100 μg/mL) of BSA or highly purified Cyt *c* from equine heart (Sigma) were added to the binding reaction.

### Protein–dsDNA docking

The 3D structures of Apaf-1-like molecules (PDB ID: 3JBT for human Apaf-1, PDB ID: 3SFZ for mouse Apaf-1, PDB ID: 5JUL for *Drosophila* Dark) were downloaded from the RCSB Protein Data Bank and the BbeApaf-J full-length structure was predicted using AlphaFold2. Protein structures were docked with ISD using Discovery Studio following software instructions. PyMol was used to visualize molecular models. Electrostatic potential was calculated using APBS in PyMol and was color-coded as −59 (red) to +59 (blue) kT/e.

### Viral DNA immunoprecipitation

HEK293T cells were transfected with 2 μg Flag-tagged Apaf-1, cGAS, or RIG-I expression vectors for 24 h, followed by HSV-1 infection for another 16 h. Cells were harvested and lysed with TAP lysis buffer. Flag M2 agaroses (Sigma-Aldrich) were added to the cell lysate and incubated overnight at 4 °C. The bead-bound immunoprecipitates were washed for 4 times with lysis buffer containing 1 M NaCl. Proteins were extracted for western blotting analysis and the DNA was extracted using QIAGEN QIAamp DNA Mini Kit. The purified DNA was used for qPCR analysis of HSV-1 DNA. For endogenous Apaf-1–DNA binding analysis, Apaf-1-associated immunoprecipitates were prepared by incubating IgG or anti-Apaf-1 (2 μg) antibody and protein G beads with cell lysates of HSV-1-infected A549 cells. DNA was extracted using QIAGEN QIAamp DNA Mini Kit before qPCR analysis for HSV-1 DNA.

### Purification of recombinant proteins

Insect cell expression of Apaf-1-like proteins was achieved with the Bac-to-Bac Baculovirus Expression System (Thermo Fisher Scientific) by following the manufacturer’s instructions. Sf9 insect cells were grown in Sf-900 II SFM at a density of 5 × 10^5^ cells per mL. Cellfectin™ II reagent was used to transfect bacmids harboring human or mouse Apaf-1 or Dark into Sf9 insect cells for recombinant baculovirus production. One liter of cells (2 × 10^6^ cells/mL) was infected with 25 mL baculovirus stock and cultured at 27 °C for large-scale expression. The infected cells were harvested after 72 h by centrifugation and resuspended in 3 volumes of Buffer A (20 mM HEPES-KOH, pH 7.5, 10 mM KCl, 5 mM MgCl_2_, 1 mM EDTA, 1 mM EGTA, 1 mM DTT). The resuspended cells were lysed in Buffer A by homogenization. His-tagged proteins were purified by affinity chromatography using Ni-NTA beads. Proteins were eluted with Buffer A containing 250 mM imidazole. The eluted Apaf-1-like proteins were stored in multiple aliquots at –80 °C.

### SRP

Analyses of nucleic acids binding and binding kinetics were performed at 25 °C on a Biacore T200 SPR instrument (Cytiva). The running buffer containing 20 mM HEPES (pH 7.5), 150 mM NaCl, 10 mM KCl, 5 mM MgCl_2_, 1 mM EDTA, 1 mM EGTA, 1 mM DTT and 0.1% (v/v) CHAPS was prepared and filtered before use. Biotinylated dsDNA HSV60, ISD, or dsRNA analogs poly (I:C) were immobilized onto flow cells Series S Sensor Chip SA (Cytiva). Insect-cell purified human or mouse Apaf-1 proteins were diluted in running buffer at different indicated concentrations and passed over adjacent target and control flow cells at a flow rate of 30 μL/min for 120 s. After 450 s dissociation, the bound analytes were removed by a 20 s wash with 20 mM NaOH. The resulting data after subtracting the control values were analyzed by fitting to the two-state reaction model using the Biacore T200 evaluation software.

### EMSA

The binding of Apaf-1-like proteins with dsDNA was monitored by EMSA. Briefly, biotinylated dsVACV70 or dsHSV60 (10 fmol or 60 fmol) was incubated with recombinant human, mouse Apaf-1 or Dark proteins at increasing amounts (0, 25, 50, 100, 200 ng) in binding buffer (20 mM HEPES pH 7.5, 5 mM MgCl_2_, 0.5 mM EGTA, 0.1% CHAPS). Reactions (20 μL) were incubated at 37 °C for 2 h before separation on 3.5%/8% native TBE gels using 0.5× TBE buffer as a running buffer. The protein−DNA complexes in the gel were transferred to a nylon membrane and blotted as instructed by the LightShift Chemiluminescent EMSA Kit (Thermo Fisher Scientific).

### Preparations of viruses and infection

HSV-1 (F strain) was provided by Dr. Chunfu Zheng (Soochow University), VACV (Western Reserve strain) was provided by Dr. Zhengfan Jiang (Peking University) and VSV-eGFP was from Dr. Xiaofeng Qing (Suzhou Institute of Systems Medicine). All were propagated and titrated in Vero cells. For in vitro infections, cells were plated at a density of 3 × 10^5^ cells in 12-well plates. The next day, the cells were infected with HSV-1, VACV or VSV for 1 h with gentle shaking. The cells were then washed and incubated in 1 mL of media containing 10% FBS. For in vivo virus infections, eight-week-old WT and *Apaf-1*^+/−^ mice were infected with VACV intraperitoneally for 16 h (2 × 10^7^ PFU per mouse) and HSV-1 intravenously for 24 h or 72 h (1 × 10^7^ PFU per mouse).

### DNA virus infection or cytoplasmic DNA stimulation of WT or *Apaf-1*^−/−^ cells and qPCR assays

To test the expression pattern of cytokines upon DNA virus infection or cytoplasmic DNA stimulation when Apaf-1 was deficient, WT and *Apaf-1*^−/−^ MEF or A549 cells were infected with HSV-1 (MOI = 3) or VACV (MOI = 3) or transfected with HT-DNA, calf thymus DNA, DNA90 and ISD at a final concentration of 3 μg/mL as indicated. Then, qRT‒PCR assays were performed using primer suggested by http://pga.mgh.harvard.edu/primerbank/. All qRT‒PCR results were normalized to the housekeeping mouse gene *Hprt* or human gene *β-actin* within each sample and compared to untreated controls to calculate the relative expression. All data are presented as the mean ± SEM of triplicates, representative of three experiments. *P* values were calculated using two-tailed unpaired Student’s *t-*test.

### RNA-seq

RNA-seq studies were performed with WT and *Apaf-1*^−/−^ MEFs transfected with or without HT-DNA (3 μg/mL) for 4 h. Total RNA was isolated using TRIZOL Reagent (Invitrogen). RNA integrity was measured using an RNA Nano 6000 assay kit of the Agilent Bioanalyzer 2100 system. Sequencing libraries were constructed using the NEBNext UltraTM RNA Library Prep Kit for Illumina (NEB) following the manufacturer’s recommendations. The poly (A)-containing mRNA molecules were purified using poly (T) oligo-attached magnetic beads. Purified libraries were quantified using a Qubit 2.0 fluorometer (Life Technologies) and validated using an Agilent 2100 Bioanalyzer to confirm the insert size. Libraries were sequenced using an Illumina HiSeq Xten platform to generate paired-end reads. The expression of transcripts was quantified as reads per kilobase of exon model per million mapped reads (RPKM). Library construction and sequencing were performed at Biomarker Corporation.

### Measurement of Cytokine Production

WT or *Apaf-1*^−/−^ MEF cells were infected with HSV-1 (MOI = 3) or transfected with DNA at a final concentration of 3 μg/mL as indicated. The concentrations of IL-6 in the culture supernatants were measured by an ELISA kit purchased from R&D Systems according to the manufacturer’s instructions.

### Generation of *Apaf-1*^−/−^, *Apaf-1*^−/−^*cGas*^−/−^, *Casp9*^−/−^ and *Rip2*^−/−^ cells

*Apaf-1*^−/−^and *Rip2*^−/−^ A549 cells were constructed using the CRISPR–Cas9 gene-editing system. The guide RNAs (gRNAs) targeting the genome sequence of target genes were cloned into the PX458 vector. The target sequences used were 5’ GCTTCAACATAGAGAAGCTC 3’ for Apaf-1 and 5’ CCCCGCAACACTAGGTCACG 3’ for RIP2. PX458 vectors containing *Apaf-1* or *RIP2* gRNAs were transfected into A549 cells using JetPRIME, and single-cell colonies of GFP^+^ cells were picked and validated at the protein expression level by western blotting assay. For *Apaf-1*^−/−^*cGas*^−/−^ and *Casp9*^−/−^ MEF cells, the gRNAs targeting the genome sequence of cGAS or Caspase-9 were cloned into the lenti-CRISPR V2 vector. The target sequences used were 5’ AGATCCGCGTAGAAGGACGA 3’ for cGAS and 5’ ATCTGGGTCTCGGCGGGATC 3’ for Caspase-9. After MEF cells were seeded, the medium was replaced with DMEM containing lentiviruses expressing Cas9 and specific gRNAs, and polybrene (8 μg/mL) (Sigma-Aldrich) was added for 48 h. Cells were then selected by puromycin (Sigma-Aldrich) for 5 days, and pooled knockout cells were generated for clonal screening and following experiments. Then the single clone was picked up for expansion with another round of puromycin selection for 2 weeks. The knockout efficiency was checked by sequencing and immunoblot analysis.

### Generation of *Rip2*^−/−^ A549 cells with stable gene expression

cDNAs encoding human RIP2 (WT, truncated or kinase-dead mutants) with N-terminal EGFP tag were generated by the standard PCR cloning strategy. For stable gene expression, RIP2 cDNAs were constructed into the pCDH-CMV-MCS-EF1-Puro vector. Lentiviruses were produced by transfecting HEK293T packaging cells with the packaging plasmids psPAX2 and pMD2.G along with the transgene in pCDH-CMV-MCS-EF1-Puro (4 μg: 3 μg: 1 μg for 6 cm dishes) using jetPRIME (polyplus). After transfection for 6 h, the medium was removed and the fresh warm medium was added. Lentiviral supernatants were collected 24 h and 48 h after transfection. *Rip2*^−/−^ A549 cells were transduced with lentiviruses, and 8 μg/mL polybrene (Sigma, Cat# H9268) was added. After 48 h, the cells were treated with puromycin (1.5 μg/mL) (Sigma-Aldrich) for one week and all live cells with strong EGFP expression were sorted by flow cytometry.

### Subcellular fractionation

Cell fractionation assays were performed according to the protocols of the NE-PER Nuclear and Cytoplasmic Extraction Kit (Thermo Scientific). The purity of nuclear and cytoplasmic extracts was assessed by immunoblotting with anti-Histone H3 (Proteintech) or anti-GAPDH antibodies (Proteintech), respectively.

For detection of Cyt *c* in cytosolic extracts, WT MEFs (2 × 10^6^) were infected with HSV-1 (MOI = 3) as indicated and resuspended in 300 μL buffer containing 150 mM NaCl, 50 mM HEPES pH 7.4, and 25 μg/mL digitonin (Sigma). The homogenates were incubated for 10 min at 4 °C on a rotary wheel and then centrifuged at 2000× *g* for 10 min. The supernatants were transferred to fresh tubes and centrifuged at 17,000× *g* for 10 min to obtain the pure cytosolic fraction for further western blotting analysis. The remaining pellets were washed twice with PBS and resuspended in 300 μL of buffer H (0.22 M mannitol, 0.07 M sucrose, 20 mM HEPES pH 7.2, 1 mM EGTA), homogenized with a Dounce homogenizer (60 strokes), and the homogenates were centrifuged at 600× *g* for 5 min to remove nuclei. Supernatants were pooled and centrifuged at 8000× *g* for 10 min, and pellets were saved as the mitochondrial (mito) fraction. ATP5A (Proteintech) was used as a mitochondrial loading control, while GAPDH (Proteintech) was probed as a cytosolic loading control.

### Cell viability assay and flow cytometry

MEF, A549, and *Apaf-1*^−/−^ A549 cells were seeded on 12-well plates 24 h before stimulation. Cells were infected with HSV-1 (MOI = 3) or VSV (MOI = 3), transfected with HT-DNA (3 μg/mL), or treated with 0.5 μM staurosporine (STS) for 8 h or 12 h. CellTiter-Lumi™ Plus Luminescent Cell Viability Assay (Beyotime) was used to measure cell viability. For flow cytometry analyses, cells were collected, washed with PBS twice, and stained using the annexin V-FITC/PI Apoptosis Assay Kit (BD) according to the manufacturer’s instructions. Stained cells were analyzed with a Beckman CytoFLEX flow cytometer, and data were processed using FlowJo software.

### Luciferase reporter assay

*cGas*^−/−^ HeLa cells seeded on 48-well plates were transfected with 100 ng of the luciferase reporter vector controlled by the NF-κB binding motif with a total of 500 ng of various expression vectors or an empty control vector. After 24 h, cells were infected with HSV-1 (MOI = 1). Cells were harvested after 12 h. The luciferase activity in the total cell lysate was detected with the Dual luciferase Reporter Assay System (Promega). A total of 2 ng of the Renilla-luciferase reporter gene was transfected simultaneously for internal control.

### Co-immunoprecipitation

HEK293T cells in 6-well dishes were transfected with a total of 4 μg DNA plasmids (3 μg HA-tagged full-length or truncated human Apaf-1 expression vectors and 1 μg Flag-tagged full-length or truncated human RIP2 expression vectors). At 24–36 h post-transfection, the whole cell extracts were prepared in TAP lysis buffer, incubated with primary antibodies at 4 °C overnight, and then incubated with Protein G Sepharose (Roche) at 4 °C for 2 h. The beads were washed three times with lysis buffer, mixed with 4× SDS loading buffer, and boiled for 10 min. Analysis was conducted using SDS‒PAGE followed by western blotting assay using the ECL protocol (Amersham Pharmacia).

### Evolutionary analysis and tree building

To generate phylogenetic trees of RIPK kinase domains across species, RIP kinase homologs of lancelets were retrieved based on the best hits of an extensive BLASTP against NCBI with *Petromyzon marinus* RIPK2 as the query. All hits were selected with the sequence identity > 30% and E value < 1e–20 and MMSeqs2 was used to remove protein redundancies (minimum sequence identity = 0.99). We further used hmmsearch 3.0 to identify the kinase domain (PF07714) of these obtained sequences, which were aligned using MAFFT (automatically determined strategy), and the final aligned 93 sequences were used to construct a phylogenetic tree by IQtree with no outgroup. iTOL was used for tree visualization and annotation.

### Statistical analysis

The data are represented as the mean ± SEM of three independent experiments. Statistical analyses were done using the software Graphpad Prism version 8.0 (GraphPad Software, Inc, La Jolla, CA, USA). The log-rank (Mantel-Cox) test was used for mice survival analysis. Unpaired two-tailed Student’s *t*-test was used for all statistical analyses.

## Supplementary information

Supplementary Information

Supplementary Table S1

## Data Availability

RNA-seq data generated during this study are deposited at the Sequence Read Archive (SRA) under access number: PRJNA951375.
