# Supplementary material for: Apaf-1 is an evolutionarily conserved DNA sensor that switches the cell fate between apoptosis and inflammation
Source: Cell Discov. 2025 Jan 21;11:4. doi: 10.1038/s41421-024-00750-4 (PMC11747288; doi:10.1038/s41421-024-00750-4)
Supplement: Supplementary file 1 — Supplementary Information [file 41421_2024_750_MOESM1_ESM.pdf]

## Supplementary Information

**a**

```
   1 MDARDHHKLR MNHRLLKRTV STHYIKGYLY EKGVLTLDDV DRIDNKATEE
  51 DKMEMLLFML PRKGDGAFQK FCSALKEKQD GTATYKEVAE KLESTVSSSE
 101 EEREFQQGSS PDVREDVARW DLLRKHRRDI ATCLDTEKTC LLLLNSDVLT
 151 DRERTVIERE ESPPERAQTL LRILPTKGTT AFHAFRQALK DQRRDDLAAL
 201 LAGEPGRVYN PVKNFAKPVS HFYTSEDARQ VNLAMEGGRF VVVLSGITGS
 251 GKTQLALWQA QLFVERHPTA VVWRLDGHDK KSFLTDKQNL LQALKEKVPT
 301 DDTQLDDCVA KALDNRKTPV LLIMDDLDDG LFLSPALLKP RKESKILLIT
 351 HRKRLQQPSN VSIPEDSYIT VNGFLKEEAV EFLRMKLQDH PADELRSLAT
 401 TFSGLPLGLA AARSYQFYAK TSVENYLTLL EKRETASKLE EKSDKWMSQF
 451 YERPEDQKAG RNLFAALRLA VSKLDQQTRS MFQLTGYMDK SKIPIVVLKE
 501 DVQDSPAMRN VALNHLVMQV EDLSLGTVEG IDNDRVLSVH EVTQLVMRLS
 551 FTEQEEKERL KKLQDILLKY FVKDNRYTKS GRLAELLQPH VGKALRYAKA
 601 VNDDGRFSLA LARLSEVYGF MNTQCGMPGN AEEHLRAAKD HIEELAKIDW
 651 RDVNYQVIEK LQAHTRGYIS IDEQGSVEEK SVKEAEVLYW KLSEASKRLP
 701 DEVFTTMIQS RVIVDQDLAL FRALGKAPAG LAESVRNKEP LTQAQYRKMV
 751 EDGIAIDVER MKEAYLPELY ASINYSLGRC FFYMKEKYAN DPAGKDSLIR
 801 SMQLAYYVCD QIKKHTGIPV LHKYLSETNA LLYLRLEEEG KSPDDLRRDI
 851 KYATKRYSEF VEGRGEYFEF GVLKKSGQDS YSLMMCQRQL VKCFNALIKL
 901 SETEEEKLAY FEQAQTECRN MITQAEKQVE TDAYGKIVER PERLAVFYNT
 951 AGRLFADDGD SKKLEEALGW FQKAYDLETE RGKEDYPLSD ALFGLAEGLV
1001 RRNHGDDLEK AEGYVGELLE LYNRKWSDKK DQIKKAQEIQ VQIAQLK*
```

**b**

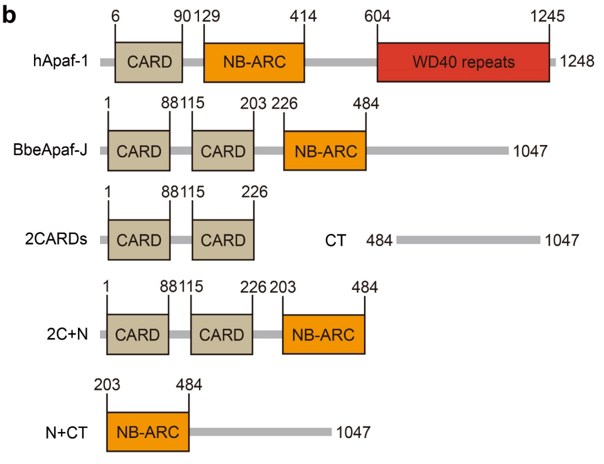


**c**

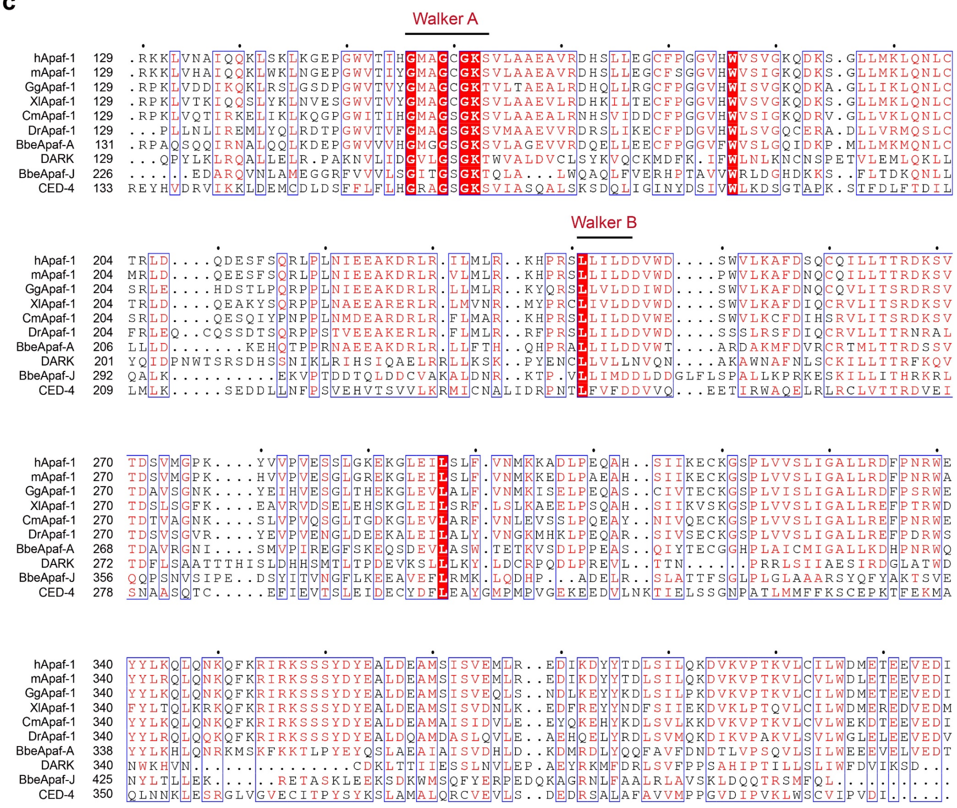


**Supplementary Fig. S1 The deduced amino acid sequence, domain structure and alignment of BbeApaf-J and related proteins.**

**a**. The amino acid sequence of BbeApaf-J. Sequences detected by MS are highlighted in red.

**b.** Domain structures of human Apaf-1 and BbeApaf-J. Numbers correspond to amino acid residues.

**c.** Multiple sequence alignment of NB-ARC domains from human, mouse, chicken (*Gallus gallus*, Gg), frog (*Xenopus laevis*, Xl), shark (*Callorhinchus milii*, Cm), zebrafish (*Danio rerio*, Dr) Apaf-1 homologs, *Drosophila* Apaf-1 homolog Dark, *C. elegans* CED-4 and lancelet Apaf-1-like proteins. The alignment was performed by using the ClustalW2 algorithm. The consensus sequence of the Walker A box and Walker B box are indicated with red.

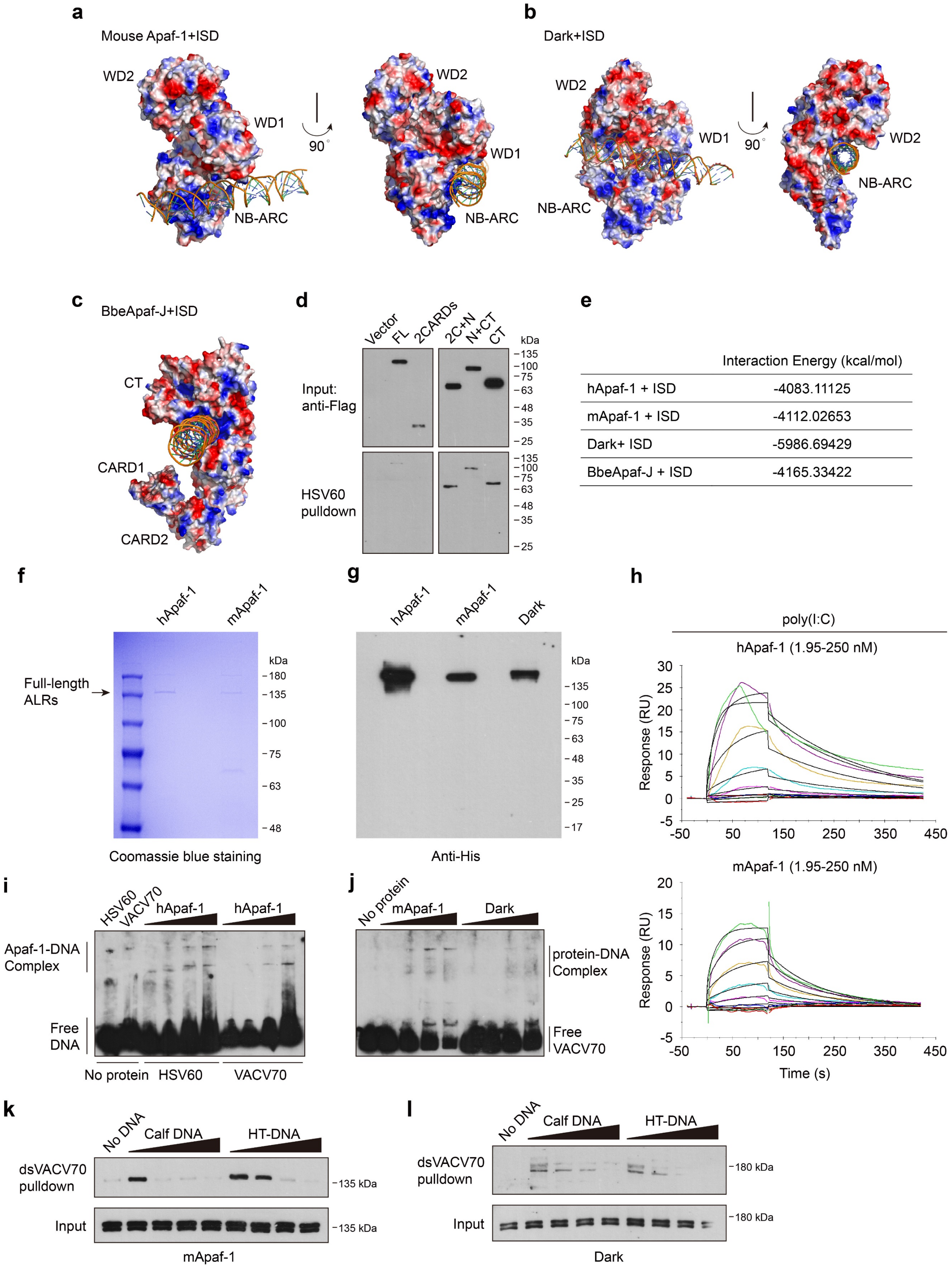


| | Interaction Energy (kcal/mol) |
|---|---|
| hApaf-1 + ISD | -4083.11125 |
| mApaf-1 + ISD | -4112.02653 |
| Dark+ ISD | -5986.69429 |
| BbeApaf-J + ISD | -4165.33422 |

**Supplementary Fig. S2 Apaf-1-like receptors directly bind to double-stranded DNA.**

**a-c**. Docking structures of the interaction of mouse Apaf-1 (PDB: 3SFZ), *Drosophila* Apaf-1 homolog Dark (PDB:5JUL) or BbeApaf-J (AlphaFold2 rank #1 models) with dsISD. Surface

electrostatics of the structures was calculated using APBS in PyMol and is color coded as −59 (red) to +59 (blue) kT/e. Blue and red, positive and negative charge, respectively.

**d**. Full-length or truncated human BbeApaf-J proteins were expressed in HEK293T cells and then incubated with streptavidin beads with biotin-HSV60. Bound proteins were analyzed by immunoblotting with anti-Flag antibodies.

**e**. The predicted interaction energy of indicated Apaf-1-like molecules-dsISD interaction were shown.

**f**. Coomassie blue staining of His-tagged human and murine Apaf-1 proteins expressed and purified from Sf9 insect cells, which were used in SPR experiments.

**g**. Immunoblotting of His-tagged human, murine Apaf-1 and Dark proteins expressed and purified from Sf9 insect cells.

**h**. The sensorgrams of chip-immobilized poly(I:C) binding to different concentrations of human or mouse Apaf-1 proteins (color lines) are shown, which are expressed in RU (response unit) versus time after subtracting the control signal. Black lines are from model fits. The concentrations of Apaf-1 proteins were 1.95, 3.9, 7.8, 15.6, 31.2, 62.5, 125, 250 nM (from bottom to top).

**i**, **j.** Electrophoretic mobility shift assay (EMSA) of purified Apaf-1-like proteins at increasing amounts (0, 25, 50, 100, 200 ng) with a low concentration of biotin-labeled dsHSV60 or dsVACV70 (10 fmol).

**k**, **l**. Pull-down competition assays using baculovirus-infected Sf9 cellular lysates with increasing amounts (0, 5, 25, 100 μg/ml) of calf thymus DNA or herring testis DNA (HT-DNA). Data are representative of three independent experiments with similar results.

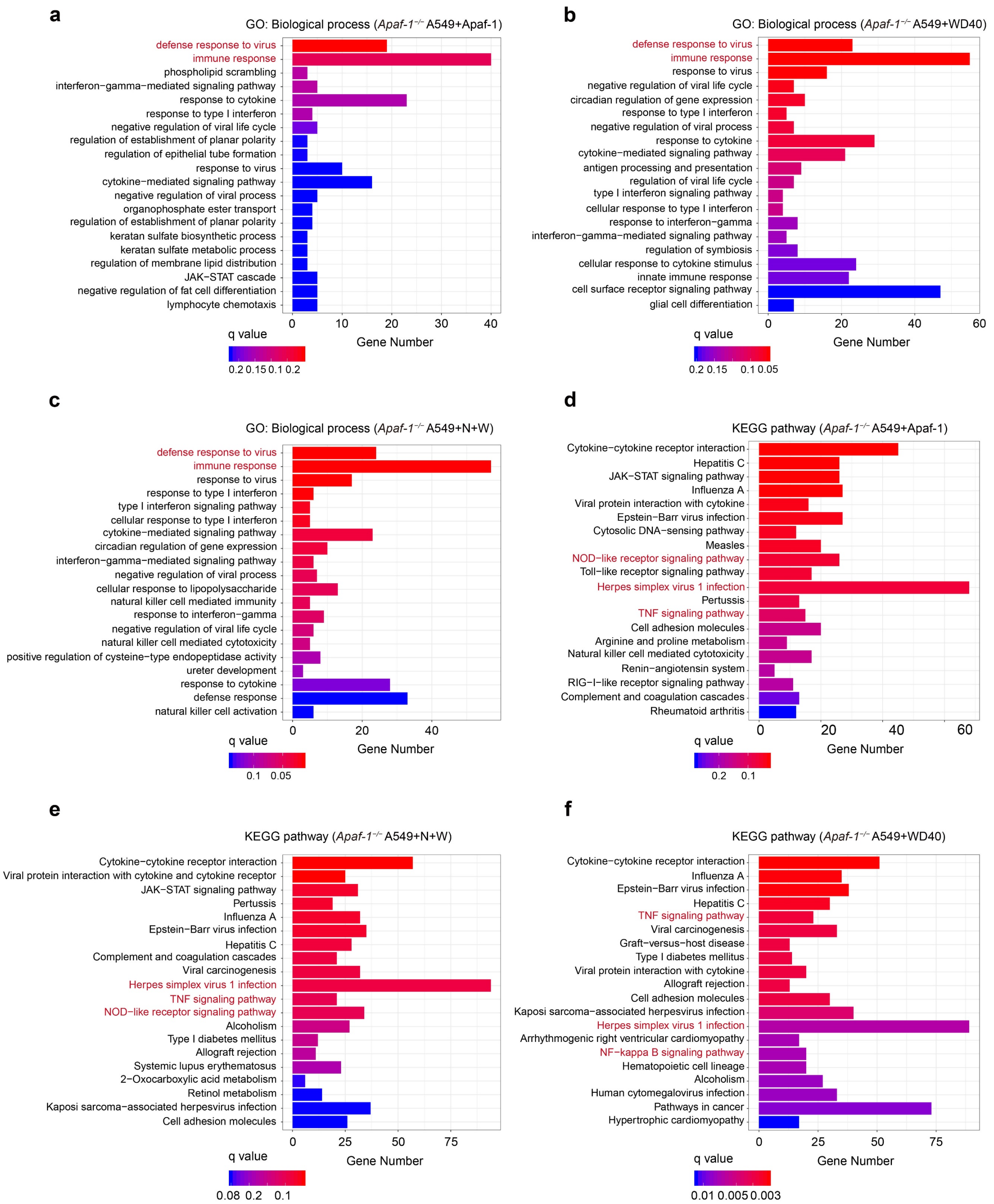


**Supplementary Fig. S3 Reconstitution of full-length or WD40 repeat domain-containing mutants of Apaf-1 in *Apaf-1*$^{///}$ A549 cells activates the innate immune response against viral infection.**

**a-c**. Gene Ontology (GO) analysis of the biological processes enriched in differentially expressed genes between *Apaf-1* $^{-/-}$ A549 cells transfected with control vectors and with full-length or the WD40 repeat domain of human Apaf-1.

**d-f**. KEGG analysis of the pathways enriched in differentially expressed genes between *Apaf-1* $^{-/-}$ A549 cells transfected with control vectors and with full-length or the WD40 repeat domain-containing mutants of human Apaf-1.

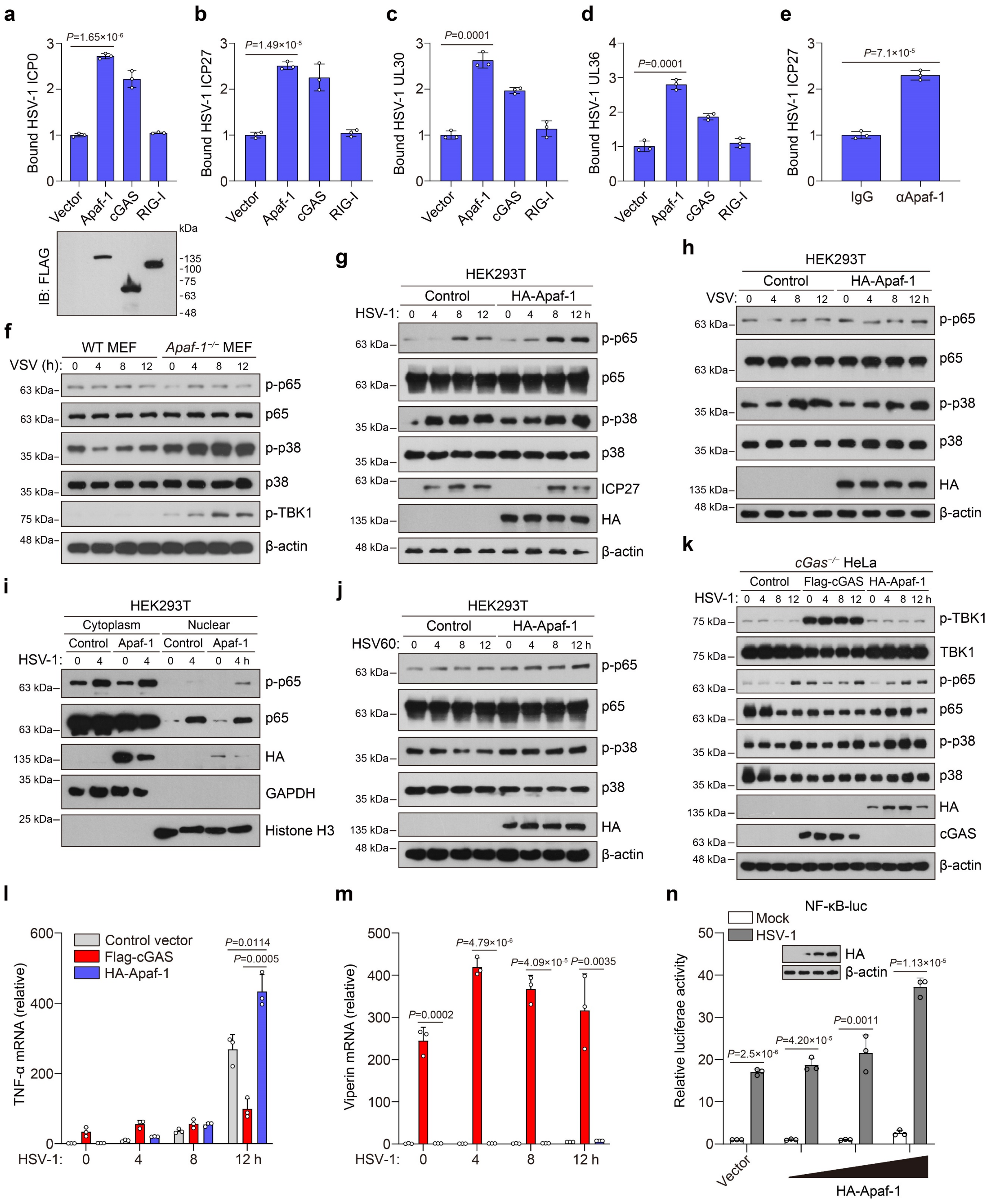


**Supplementary Fig. S4 Apaf-1 mediates DNA- or HSV-1-induced NF-κB activation independently of cGAS and STING.**

**a-d**. HEK293T cells expressing indicated FLAG fusion protein were infected with HSV-1, immunoprecipitated and analysed by qRT–PCR to detect bound HSV-1 DNA. HSV-1 *ICP0*,

*ICP27*, *UL30* and *UL36* genes were quantified by qRT–PCR. The data are presented as fold increase over vector.

**e**. qRT–PCR analyses of HSV-1 DNA bound by endogenous Apaf-1 in A549 cells.

**f**. Western blot analysis of the phosphorylation of p65 , p38 and TBK1 in WT and *Apaf-1*$^{-/-}$ MEFs infected with VSV (MOI = 3) for the indicated times.

**g**. HEK293T cells expressing control vectors or HA-Apaf-1 were infected with HSV-1 (MOI = 1) for the indicated times. p65, p38 phosphorylation, and the HSV-1 protein ICP27 were monitored by immunoblotting.

**h**. HEK293T cells expressing control vectors or HA-Apaf-1 were infected with VSV (MOI = 1) for the indicated times. The phosphorylation of p65 and p38 was monitored by immunoblotting.

**i**. HEK293T cells expressing control vectors or HA-Apaf-1 were infected with HSV-1 (MOI = 1) for 4 h, and then cell lysates were separated into cytosolic and nuclear fractions. Each fraction was concentrated and subjected to immunoblotting with the indicated antibodies.

**j**. HEK293T cells expressing control vectors or HA-Apaf-1 were stimulated with 3 μg/mL HSV60 for the indicated times, and then phosphorylated (p-) p65 and p38 were determined by immunoblotting.

**k-m**. *cGas*$^{-/-}$ HeLa cells expressing control vectors, Flag-cGAS and HA-Apaf-1 were infected with HSV-1 (MOI = 3) for the indicated times. The phosphorylation of TBK1, p65, and p38 and the expression of *TNF-α* and *Viperin* were assessed by immunoblotting (**k**) or qRT–PCR (**l** and **m**).

**n**. *cGas*$^{-/-}$ HeLa cells expressing increasing amounts of Apaf-1 plasmids together with NF-κB

promoter-driven luciferase reporters were infected with HSV-1 (MOI = 3) for 12 h. NF-κB activation was assessed by luciferase reporter activity.

Data in (**l-n**) are presented as the mean ± SEM of triplicates, and *P* values were calculated using two-tailed unpaired Student's *t* test.

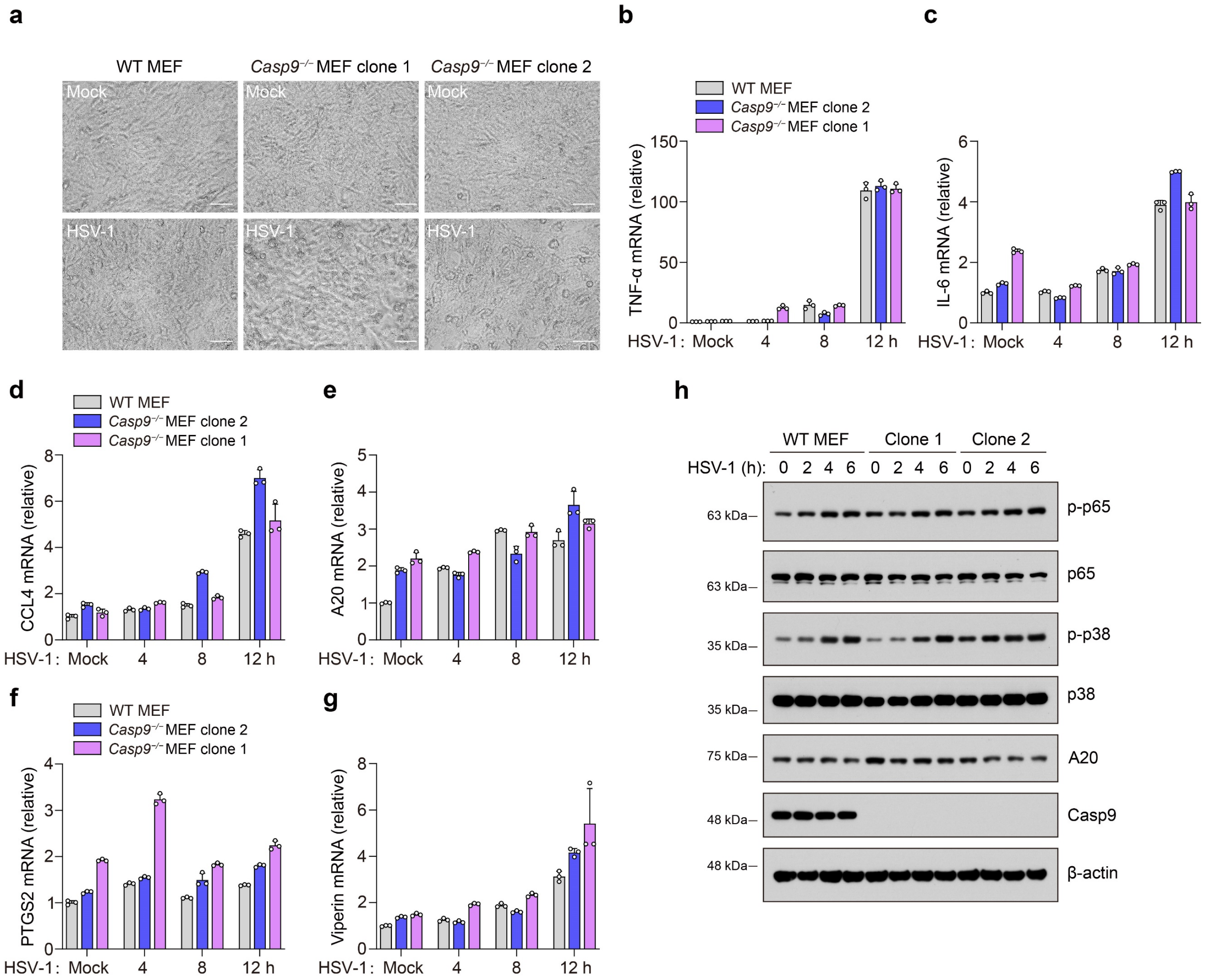


**Supplementary Fig. S5 Caspase-9 is dispensable for DNA virus infection-induced inflammatory responses.**

**a**. WT and *Casp9*$^{-/-}$ MEF cells were infected with HSV-1 (MOI = 1.5) and analyzed 12 h later, their morphology was observed by phase-contrast microscopy. Scale bars, 100 µm.

**b-g**. WT and *Casp9*$^{-/-}$ MEF cells were infected with HSV-1 (MOI = 1.5) as indicated and the expression of *TNF-α, IL-6, CCL4, A20*, *PTGS2* and *Viperin* was measured by qRT-PCR.

**h**. Western blot analysis of the phosphorylation of p65, p38 and the expression of A20 in WT and *Casp9*$^{-/-}$ MEF cells infected with HSV-1 (MOI = 3) at the indicated times.

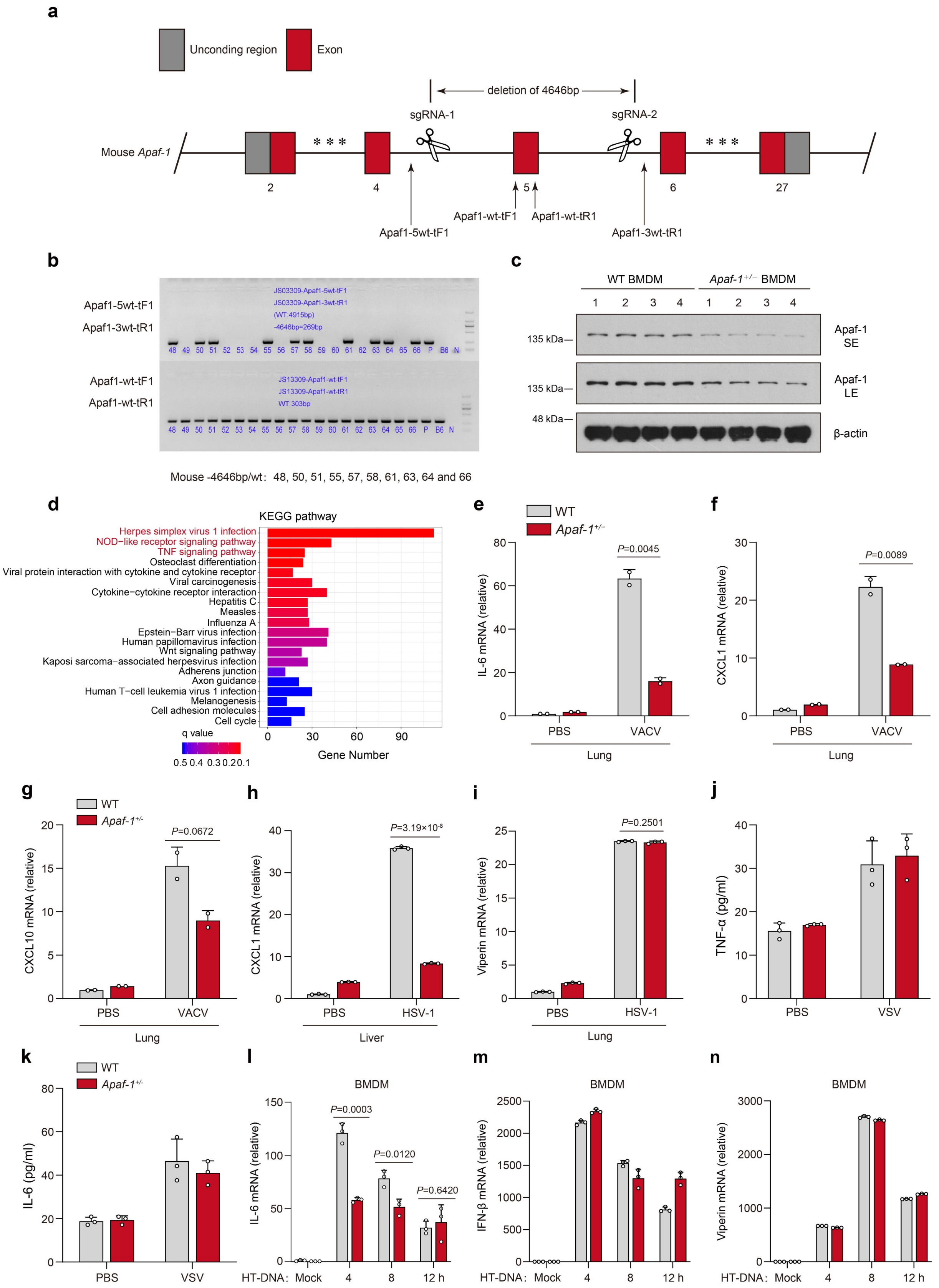


**Supplementary Fig. S6 Apaf-1 is required for DNA virus or DNA-induced inflammatory responses *in vivo* and *ex vivo*.**

**a**. Generation of *Apaf-1* $^{+/-}$ mice by CRISPR-Cas9-mediated genome editing. Two gRNAs matching the sequences flanking exon 5 of the Apaf-1 gene were used to achieve deletion of a large genomic fragment containing exon 5 (4646 bp).

**b**. PCR genotyping of *Apaf-1* $^{+/-}$ mice using the indicated primers.

**c**. Western blot analysis of Apaf-1 in primary WT and *Apaf-1* $^{+/-}$ BMDMs from different mice. SE, short exposure; LE, long exposure.

**d**. KEGG analysis of the pathways enriched in differentially expressed genes in the lungs of wild-type (WT) or *Apaf-1* $^{+/-}$ mice infected with VACV ($2\times10^7$ PFU per mouse) for 16 h.

**e-g**. qRT–PCR analysis of *IL-6*, *CXCL1* and *CXCL10* mRNA levels in the lungs from WT and *Apaf-1* $^{+/-}$ (n = 2 per group) 8-week-old mice infected with VACV ($2\times10^7$ PFU per mouse) for 16 h.

**h**, **i**. qRT–PCR analysis of *CXCL1* and *Viperin* mRNA levels in the livers or the lungs from WT and *Apaf-1* $^{+/-}$ (n = 3 per group) 8-week-old mice infected with HSV-1 ($1\times10^7$ PFU per mouse) for 24 h.

**j**, **k**. WT and *Apaf-1* $^{+/-}$ mice (n = 3 per group) were infected intravenously with VSV ($1\times10^7$ PFU per mouse) for 24 h, and then sera were collected. The levels of TNF-α and IL-6 were measured by ELISA.

**l-n**. Primary BMDMs were transfected with HT-DNA (1 μg/mL) for the indicated time points followed by measurements of *IL-6*, *IFN-β* and *Viperin* mRNA levels by qRT–PCR. qRT–PCR results were normalized to the housekeeping gene *Hprt* within each sample, and compared to untreated controls to calculate the relative expression. All data are presented as mean ± SEM of triplicates (n = 3) and *P* values were calculated using two-tailed unpaired Student's *t* test.

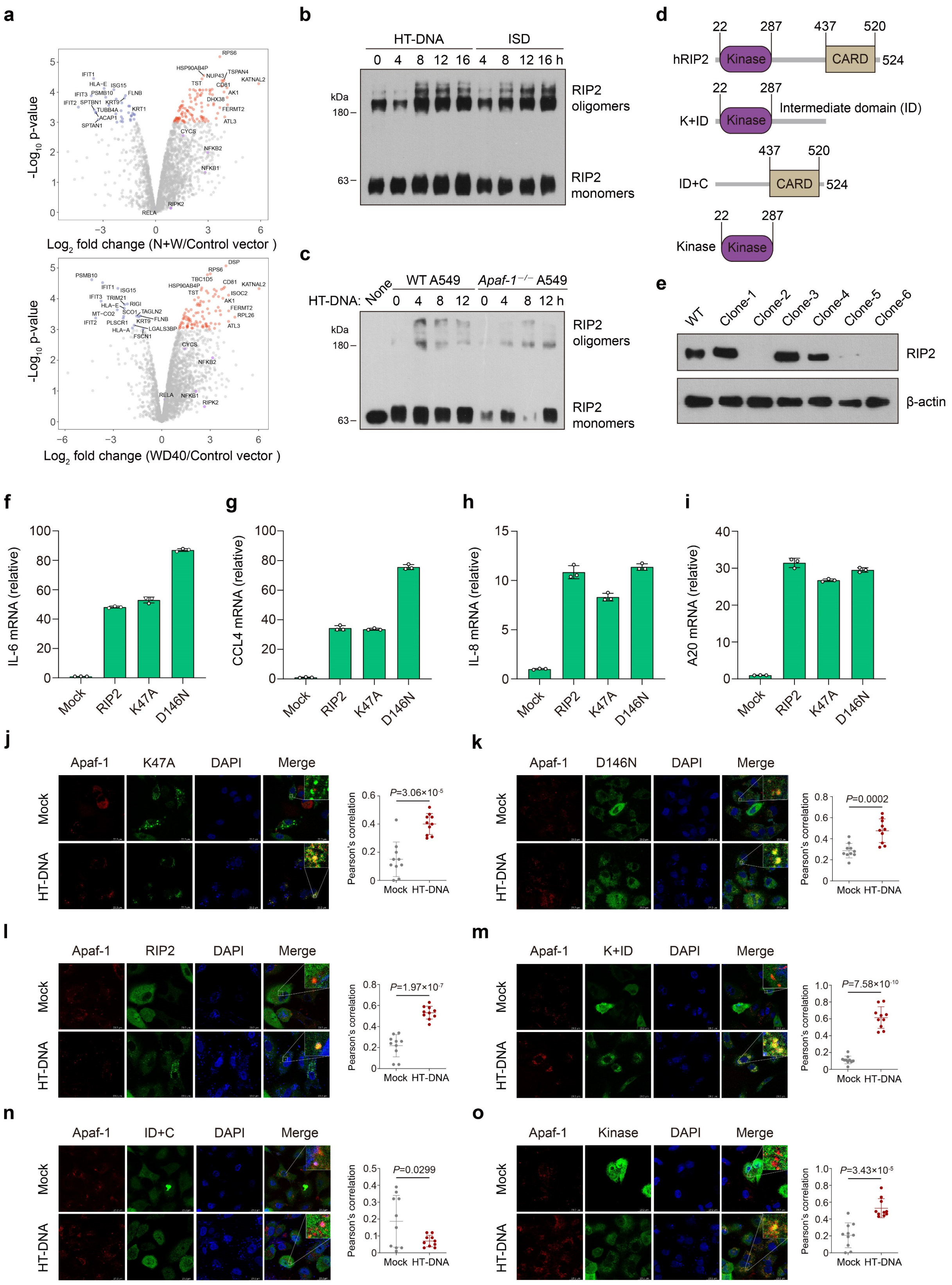


**Supplementary Fig. S7 Apaf-1 recruits RIP2 to promote its oligomerization upon cytoplasmic DNA sensing.**

**a**. *Apaf-1*$^{-/-}$ A549 cells were transfected with HA-tagged control vectors or HA-tagged WD40 domain-containing mutants of Apaf-1 for 24 h and then infected with VACV (MOI = 3) for another 2 h. The cell lysates were subjected to anti-HA IP and analyzed by LC-MS/MS. The volcano plot depicts proteins enriched in the HA-N+W IP and HA-WD40 IP.

**b**. Primary mouse ear fibroblasts were transfected with HT-DNA (3 μg/mL) and ISD (3 μg/mL) for the indicated time points. Cell lysates treated with the cross-linking reagent BS3 were analysed by immunoblotting.

**c**. WT and *Apaf-1*$^{-/-}$ A549 cells were transfected with HT-DNA for the indicated time points. The oligomerization of endogenous RIP2 was visualized by immunoblotting after treatment with the cross-linking reagent BS3.

**d**. Domain structures of human RIP2 and its truncated mutants.

**e**. Western blot analysis of RIP2 in WT and several RIP2-targeted A549 clones.

**f-i**. *Rip2*$^{-/-}$ A549 cells stably expressing EGFP-RIP2, EGFP-RIP2 K47A or EGFP-RIP2 D146N were transfected with HT-DNA (3 μg/mL) for 12 h, the expression of *IL-6*, *CCL4*, *IL-8* and *A20* was measured by qRT−PCR.

**j-o**. *Rip2*$^{-/-}$ A549 cells stably expressing EGFP-tagged RIP2 kinase dead mutants (K47A and D146N), full-length or truncated mutants of RIP2 were transfected with mCherry-Apaf-1 for 24 h, and then stimulated with HT-DNA (3 μg/mL). At 12 h post-transfection, cells were fixed, stained with DAPI and imaged by confocal microscopy. Scale bars, 23.2 μm. Pearson's correlation coefficients of Apaf-1 and indicated full-length or RIP2 mutants in 10 cells from different fields of view were used to quantify co-localization.

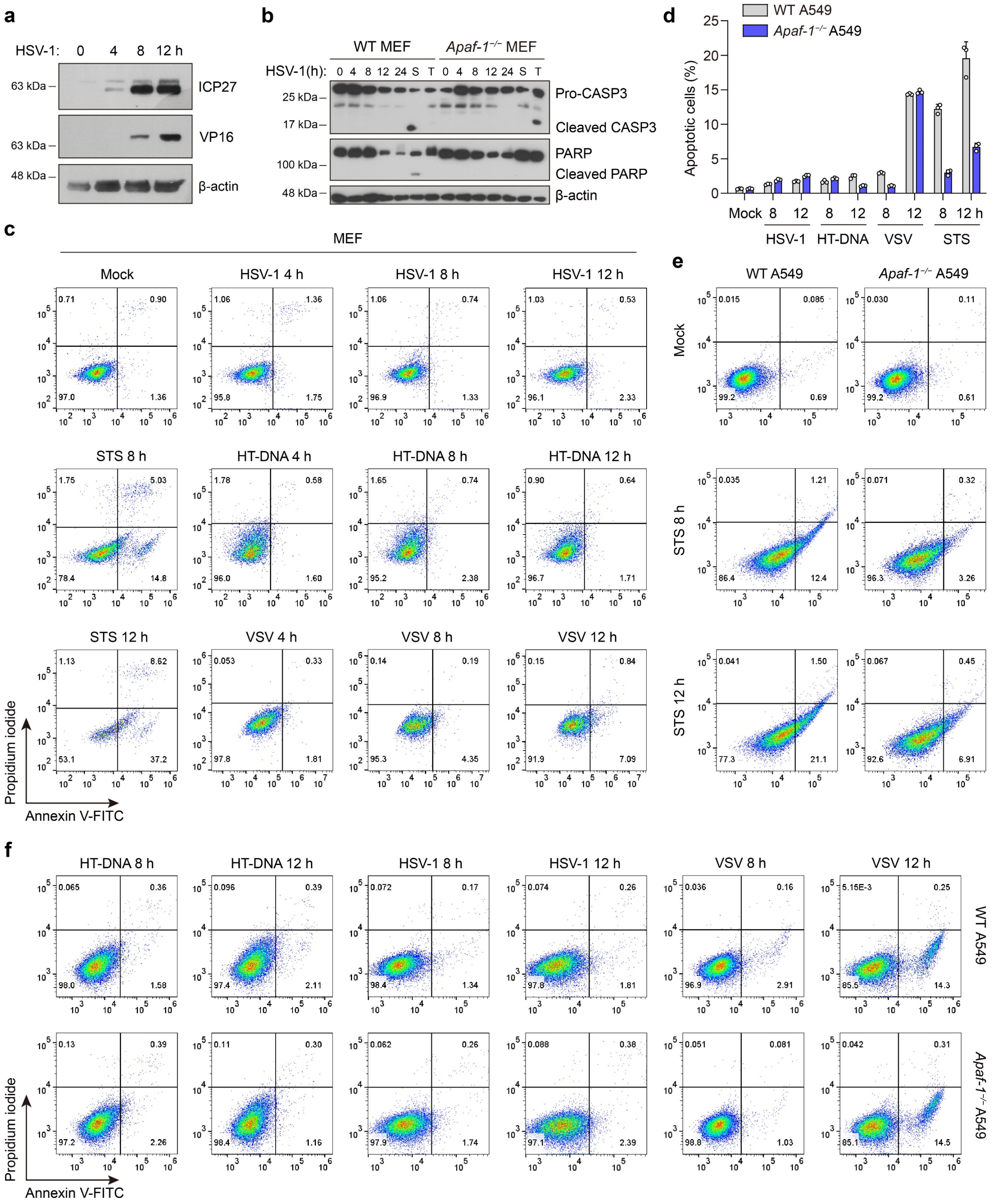


**Supplementary Fig. S8 HSV-1 infection and HT-DNA transfection do not trigger apoptotic cell death in either MEF or A549 cells.**

**a**. Western blot analysis of the HSV-1 proteins ICP27 and VP16 in MEF cells infected with HSV-1 (MOI = 3) at the indicated times.

**b**. WT and *Apaf-1*$^{-/-}$ MEFs were infected with HSV-1 (MOI = 3) as indicated or stimulated with staurosporine (S) or TNF-α (T) for 12 h. The cleavage of caspase-3 and PARP was analyzed by immunoblotting analysis.

**c**. Representative flow cytometry plots of MEF cells treated with HSV-1 (MOI = 3), HT-DNA (3 μg/mL), VSV (MOI = 3) or staurosporine (STS) at the indicated times and then were stained with propidium iodide and annexin V-FITC.

**d**. WT and *Apaf-1*$^{-/-}$ A549 cells were treated with HSV-1 (MOI = 3), HT-DNA (3 μg/mL), VSV (MOI = 3) or staurosporine (STS) as indicated and then the proportions of apoptotic cells are summarized.

**e**, **f**. Representative flow cytometry plots of WT and *Apaf-1*$^{-/-}$ A549 cells treated with HSV-1 (MOI = 3), HT-DNA (3 μg/mL), VSV (MOI = 3) or staurosporine (STS) at the indicated times, and then were stained with propidium iodide and annexin V-FITC.

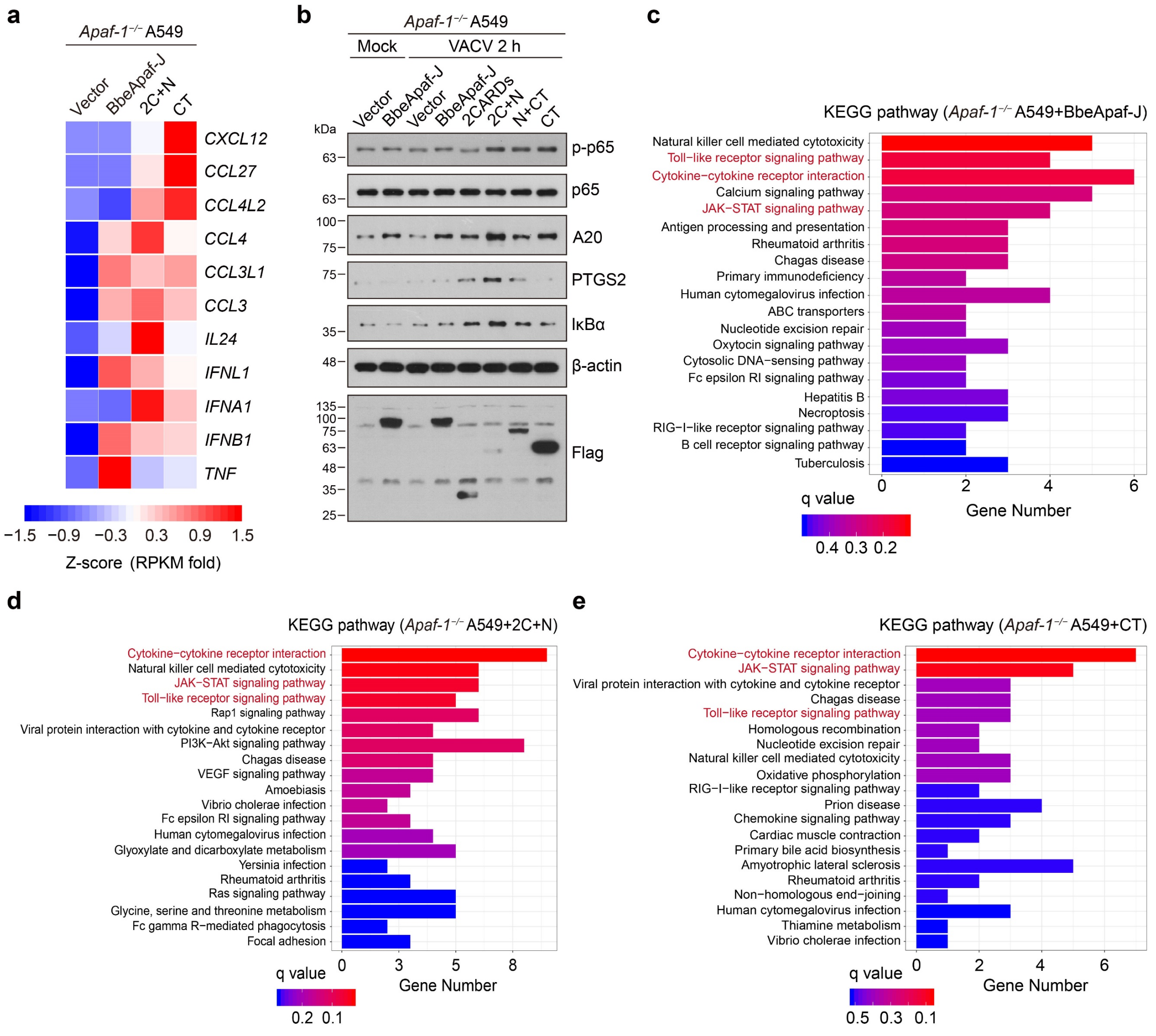


**Supplementary Fig. S9 BbeApaf-J is a conserved DNA sensor that activates inflammatory responses.**

**a**. Heatmap of RNA-seq analysis of proinflammatory cytokines and chemokines in *Apaf-1* $^{-/-}$ A549 cells reconstituted with full-length or truncated BbeApaf-J proteins for 24 h and then infected with VACV for another 16 h.

**b**. For complementation, Flag-tagged full-length or truncated BbeApaf-J proteins were expressed in *Apaf-1* $^{-/-}$ A549 cells for 24 h and then infected with VACV for another 2 h. Western blot analysis of NF-κB signaling activation is indicated.

**c-e**. KEGG analysis of the pathways enriched in differentially expressed genes between *Apaf-*

*1* $^{-/-}$ A549 cells reconstituted with control vectors and with full-length BbeApaf-J (**c**), BbeApaf-J-2CARDs+NB-ARC (**d**) or CT domain (**e**) alone.

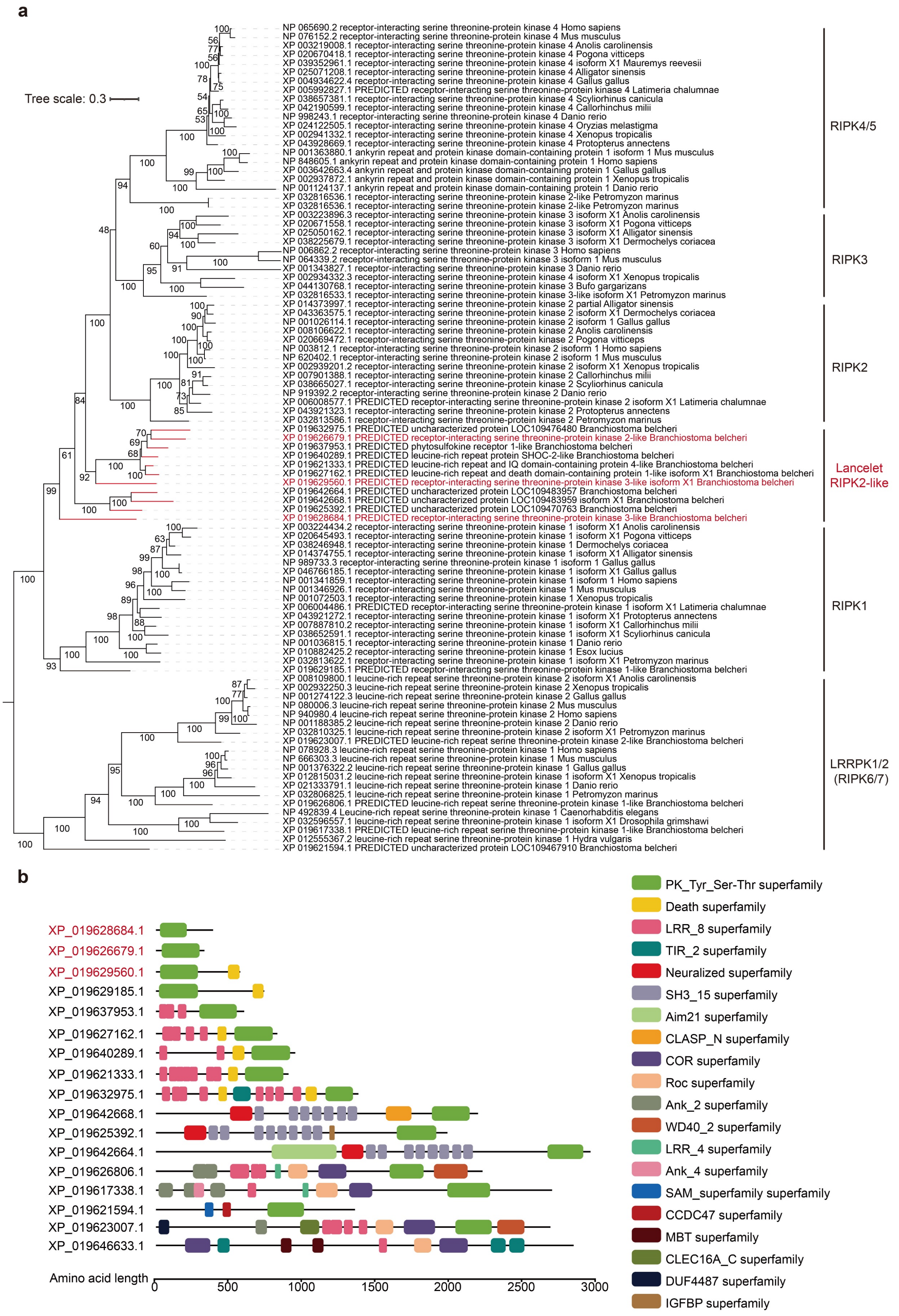


**Supplementary Fig. S10 Evolutionary analyses of the RIP kinase (RIPK) family across**

**species.**

**a**. Maximum likelihood (ML) phylogenetic tree generated by IQtree of RIPK1–7 kinase domains from indicated metazoans. The lancelet RIPK2-like clades are highlighted with red. Numbers at nodes indicate bootstrap values.

**b**. Domain structures of expanded lancelet RIPK proteins are shown approximately to scale. Three lancelet RIPK2 homologs having only one kinase domain or the classical Kinase-Death domain architecture are highlighted with red.
